# Application of Natural Functional Additives for Improving Bioactivity and Structure of Biopolymer-Based Films for Food Packaging: A Review

**DOI:** 10.3390/polym16141976

**Published:** 2024-07-10

**Authors:** Natalia Revutskaya, Ekaterina Polishchuk, Ivan Kozyrev, Liliya Fedulova, Valentina Krylova, Viktoriya Pchelkina, Tatyana Gustova, Ekaterina Vasilevskaya, Sergey Karabanov, Anastasiya Kibitkina, Nadezhda Kupaeva, Elena Kotenkova

**Affiliations:** 1 Department of Scientific, Applied and Technological Developments, V. M. Gorbatov Federal Research Center for Food Systems of the Russian Academy of Sciences, Talalikhina st., 26, 109316 Moscow, Russia; n.revuckaya@fncps.ru (N.R.); iv.kozirev@fncps.ru (I.K.); v.krylova@fncps.ru (V.K.); t.gustova@fncps.ru (T.G.); 2 Experimental Clinic and Research Laboratory for Bioactive Substances of Animal Origin, V. M. Gorbatov Federal Research Center for Food Systems of the Russian Academy of Sciences, Talalikhina st., 26, 109316 Moscow, Russia; e.politchuk@fncps.ru (E.P.); l.fedulova@fncps.ru (L.F.); v.pchelkina@fncps.ru (V.P.); e.vasilevskaya@fncps.ru (E.V.); s.karabanov@fncps.ru (S.K.); a.kibitkina@fncps.ru (A.K.); nvkupaeva@yandex.ru (N.K.)

**Keywords:** active packaging, plant antioxidants, bacteriocins, antimicrobial compounds, organic nanoparticles, starch, chitosan, alginate, cellulose, protein

## Abstract

The global trend towards conscious consumption plays an important role in consumer preferences regarding both the composition and quality of food and packaging materials, including sustainable ones. The development of biodegradable active packaging materials could reduce both the negative impact on the environment due to a decrease in the use of oil-based plastics and the amount of synthetic preservatives. This review discusses relevant functional additives for improving the bioactivity of biopolymer-based films. Addition of plant, microbial, animal and organic nanoparticles into bio-based films is discussed. Changes in mechanical, transparency, water and oxygen barrier properties are reviewed. Since microbial and oxidative deterioration are the main causes of food spoilage, antimicrobial and antioxidant properties of natural additives are discussed, including perspective ones for the development of biodegradable active packaging.

## 1. Introduction

Food packaging is important for the storage and transportation of products as well as ensuring their safety and quality by protecting from contamination and spoilage [1]. Currently, the packaging material market is largely represented by oil-based plastics, the production of which has grown significantly worldwide [2]. Plastics have a low biodegradability [3], and agricultural plastic waste reuse and recycling are very low [4]. The widespread use of plastic leads to accumulation and adverse effects on the environment and human health [5]. Therefore, there is a growing interest in biodegradable packaging materials [6,7].

The global trend towards conscious consumption plays an important role in consumer preferences regarding both the composition and quality of food and packaging materials, including sustainable ones [8,9,10,11]. This opens up a new field of activity for researchers: the development of biodegradable active packaging materials with specified functional properties (for example, antimicrobial or antioxidant), which would help to reduce both the negative impact on the environment due to a decrease in the use of oil-based plastics and the amount of synthetic preservatives/antioxidants used in food products that have an adverse effect on human health [12,13,14,15,16].

The development of biodegradable active packaging is a research area of current interest [17,18]. Over the past decade, many research projects have been developed within the framework of international funding programs, the most ambitious being Horizon 2020 and Horizon Europe. The projects focused on solving global problems and the topic of adaptation to climate change in the first place. This topic includes dozens of areas in which projects in the field of biopolymer packaging materials have a special place. Selection of component ratios (the ratio of biopolymer, plasticizer and other components) and determination of blend composition for improving structural, mechanical and other characteristics of the packaging material are intensively studied [19,20,21,22,23]. Immobilization of synthetic commercial substances, mixtures and/or mono-additives, such as antioxidants, acids, etc., in packaging matrices could improve biological activity but could be poisonous and hazardous to health [24,25,26]. Therefore, natural biologically active substances of microbial, plant and animal origins are in high demand [27,28,29,30]. This review also discusses relevant natural functional additives for improving bioactivity of biopolymer-based films.

## 2. Biopolymers for Food Packaging

**Figure 1:**
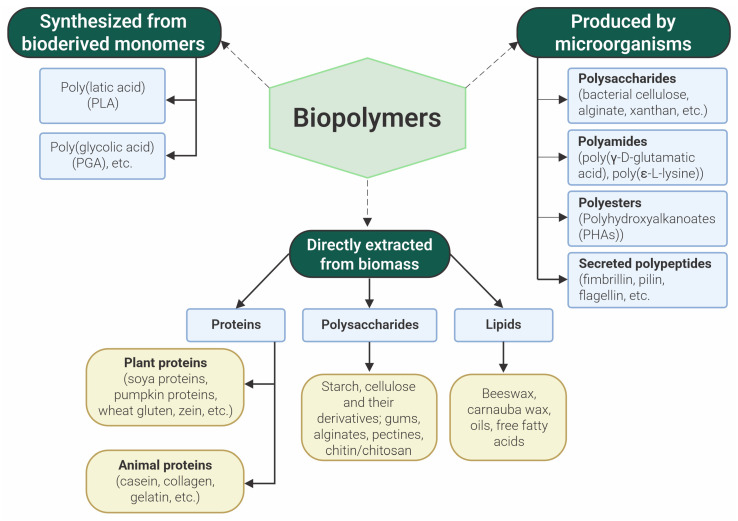
Schematic classification of biopolymer types.

The use of biopolymers has gained popularity in food packaging recently. From the perspective of environmental sustainability, it can be stated that biopolymers serve as environmentally friendly packaging materials [31]. Biopolymers are categorized into three groups based on their production method and source of origin: synthesized from bioderived monomers, extracted from biomass and produced by microorganisms [32] (Figure 1). Natural polysaccharides (starch, cellulose, chitosan, etc.) and animal proteins (collagen, gelatin, etc.) are commonly utilized as packaging materials [23] due to their wide availability and the ability to create packaging materials with specific characteristics from them.

In the production of biodegradable food packaging, special attention is paid to starch. Starch is the second most abundant organic substance found in nature and serves as the main storage carbohydrate in plants [33]. Starch is a good candidate for making biopolymer film due to its structure and crystallinity caused by hydrogen bonds, which help it be easily converted into a thermoplastic material [34]. Most published works related to starch focused on wheat, corn, potato and rice starches due to their commercial importance [33]. However, it is known that under specially adapted cultivation conditions, microalgae are capable of accumulating starch content up to 40–45% of dry weight [35], which makes them promising candidates for the production of this biopolymer. Quite promising biopolymers are polyhydroxyalkanoates (PHAs), which are biosynthesized by fermentation of sugars and lipids by a wide range of microorganisms [36,37]. Besides being biodegradable, biocompatible and renewable, PHAs are known for properties such as high tensile strength, printability, good UV resistance, grease and oil resistance, making them suitable for the food packaging industry [36].

For many years, researchers have been developing active packaging films based on natural biopolymers. However, the competitiveness of these materials still lags behind petroleum-based plastics due to insufficient mechanical properties. These shortcomings can be overcome, and furthermore, functional additives can enhance not only the activity but also the physical and mechanical properties of the films.

## 3. Methods to Make Biopolymer Films Bioactive

The incorporation of bioactive compounds into packaging materials enables the development of biopolymer films with antioxidant [38,39], antimicrobial [40,41,42] and barrier properties [43,44], thereby enhancing the safety and quality of food products. Several techniques for integrating natural functional additives into film-forming materials have been documented in the scientific literature [45]. Some of these methods include the following:

– Incorporation of bioactive substances in the film during the production process by mixing them with a film-forming solution [40,46,47];
– Grafting bioactive compounds onto biopolymer chains [48,49];
– Functionalization by bioactive groups (carboxylic, phenolic, amino groups, etc.) [50], for example, by aminolysis [51];
– Enzyme immobilization [52,53];
– Encapsulation of bioactive compounds with the formation of micro- or nanoparticles. Subsequently, during the film production process, such particles are included in its composition by direct mixing with a film-forming biopolymer solution [44,54,55,56,57] or by spraying them onto a biopolymer film [43,58].

When incorporating natural functional compounds into biopolymer materials, it is necessary to consider their compatibility as well as the concentration proportions between them [45,59].

## 4. Plant Additives

Plants have received greater attention as they contain elevated concentrations of components that possess strong antioxidant activities and can lead to both reduction in nutrient oxidation and microbial spoilage [60,61]. Essential oils and plant extracts have become the main alternative to synthetic additives [62]. Emulsions, mono-antioxidants or plant wastes are also used as components of biodegradable films for the creation of active packaging.

### 4.1. Essential Oils

Essential oils contain secondary metabolites of plants—aromatic and volatile oil liquids (almost insoluble in water)—which include monoterpenes and sesquiterpene hydrocarbons, phenolic compounds and various volatile organic compounds [63]. Essential oils could include terpenes (p-cymene, terpinene, limonene, sabinene, pinene, etc.), hydrocarbons consisting of several isopropene units, terpenoids (geraniol, menthol, linalool, citronellol, carvone, thymol, carvacrol, geranyl acetate, eugenyl acetate, geranal, neral, 1,8-cineole, etc.) and aromatic compounds—phenylpropanoids (cinnamon aldehyde, havicol, eugenol, estragol, methylcinnamate, etc.) [64]. The chemical composition of essential oils depends on variety, geographical origin, part of the plant used, age, season, harvest conditions, extraction method and solvent [65].

There are several ways of essential oil production: pressing, fermentation, enflerage or extraction. For industrial production, the method of water steam distillation is most often used [66], especially for extraction from aromatic herbs and spices [67]. Hydrodistillation involves the complete immersion of plant raw materials in water followed by boiling and allows isolating compounds insoluble in water [68]. Hydrodiffusion extraction is a type of steam distillation, differing only in the way of steam introduction. It is fed from above the plant material. Hydrodiffusion extraction can also be produced under low pressure or vacuum [69]. Subcritical water extraction applies water in a liquid state under conditions of a high temperature range (from 100 to 374 °C) at a critical pressure (1–22.1 MPa), which makes it possible to obtain bioactive compounds with minimal destruction and high activity [70]. Using liquid carbon dioxide at low temperature and high pressure or hexane allows for higher yield of active components during extraction. However, this method is much more expensive [71]. Innovative alternative extraction methods include ohmic hydrodistillation (electric heating), microwave hydrodistillation, solvent-free double-cooled microwave extraction, ultrasonic supercritical carbon dioxide extraction and ultrasonic extraction, which reduce the time and amount of energy consumed as well as improve the quality of the obtained essential oil [72,73,74,75,76,77].

Most essential oils demonstrate a wide range of antimicrobial activity against bacteria and fungi [78] due to the hydrophobicity and hydrophilic functional groups (hydroxyl groups of phenolic compounds) in the chemical structure of compounds, which can disrupt the metabolism of microorganisms [79,80,81]. Also, essential oils have antioxidant properties [82] due to terpenes and phenols such as thymol, carvacrol and eugenol, phenolic acids (rosemary acid) and monocyclic hydrocarbons such as terpinolene and γ-terpinene [83,84,85,86,87]. Rosemary, oregano, cloves, thyme, cinnamon, ginger, garlic, citrus fruits, etc., are some of the most popular sources of essential oils [88].

Essential oils are introduced into biopolymer and other films by emulsification or homogenization as any lipophilic substance [89,90]. Effectiveness depends on the type and concentration of oil, hydrophobicity, particle size and stability of the emulsion [80]. The disadvantages of essential oils are susceptibility of active components to polymerization and oxidation during the shelf life from external (light, temperature, humidity, microbial contamination, etc.) and internal (acidity, water activity of food products, etc.) factors, which can disrupt their functionality [91]. Ability to volatilize and break down during the packaging process (thermal effects, high shear rates) are also serious problems [92].

Essential oils affect organoleptics and change the transparency of edible films because they are poorly dispersed in water-based solutions [93,94]. Addition of essential oil into the film leads to an increase in thickness, while pores and roughness may be observed [95,96,97]. The solubility of films in water and swelling decrease due to an increase in the hydrophobicity of films and the formation of new intermolecular bonds [98]. The changes in structural and mechanical parameters almost had a common tendency: a decrease in the tensile strength (TS) and an increase in elongation at break (EAB), which could be linked with uneven distribution of essential oils and insufficient density of the biopolymer matrix [99,100,101,102]. Emulsifiers, plasticizers and surfactants that are rich in hydroxyl groups can be added to the film-forming solution (FFS) for better distribution of the components and to avoid the fragility of the film [103,104]. Water vapor permeability (WVP) could also be reduced due to the uneven structure of the films [101,105]. Color parameters are also changed (e.g., elevated yellowness (B*)), which are usually related to the native color of the essential oils [100,106,107,108]. Antioxidant properties were also elevated and investigated by various standard methods, such as TEAC, ABTS, FRAP, DPPH and TBARS, to assess the degree of lipid oxidation [95,96,98,99,100,101,102,103,104,107,108]. Antimicrobial activity was also demonstrated against *E. coli*, *S. enterica*, *B. cereus*, *S. aureus*, *S. Typhimurium*, *L. monocytogenes*, *P. aeruginosa*, *Candida albicans* and *parapsilosis*, etc. [95,96,98,99,100,101,102,103,104,107,108]. Gram-negative bacteria were noted to be more resistant to essential oils due to the presence of an external complex and a dense lipopolysaccharide membrane surrounding the cell wall [109].

### 4.2. Emulsions

Emulsions are oil/water systems with surfactants [110]. The application of emulsions, microemulsions and nanoemulsions can improve the functional characteristics of the film solution and increase the particle distribution [111]. Active nano substances are able to bind with many biological molecules with greater efficiency, thereby increasing antimicrobial and antioxidant properties [112]. Low thermal stability is one of the disadvantages of emulsions [104].

Surfactants have hydrophilic and hydrophobic properties and are added to emulsions to increase stability [104]. As a component of films with emulsions, surfactants and emulgators can reduce surface tension; improve wettability and adhesion of coating solutions [113]; and reduce moisture loss [114,115]. Water resistance and mechanical properties of the films can be improved by including essential oils and hydrophilic surfactants [116,117]. However, surfactants can negatively affect the antibacterial activity of the active components [90].

Microemulsions are more transparent, homogeneous and thermodynamically stable [118]. They are small droplets ranging in size from 10 to 100 nm and contain additional surfactants and/or solvents [94]. The low interfacial tension allows increasing the reactivity of active substances (essential oils) by expanding the surface area and simplifying the preparation of microemulsion without spending a lot of mechanical effort [119]. Nanoemulsions consist of particles with a diameter of 0.1 to 100 nm, have the same advantages as microemulsions, can improve physico-chemical properties of films and possess a less-pronounced effect on organoleptic characteristics [110]. The advantages of nanoemulsions include higher kinetic stability and a larger surface area ratio, which reduce the release rate [120]. Due to the bound moisture in nanoemulsions, they are considered self-preservative antimicrobials [121]. However, they have a low stability in an acidic environment and require a large amount of energy for production [11]. Pickering emulsions can be used as a stabilizer instead of surfactants [122]. Compared with traditional emulsions, a solid layer of micro- and nanoparticles (silicon dioxide, clay materials, metal and metal oxide nanoparticles, calcium and carbon particles, etc.) is formed between the aqueous and oil phases [123]. They are characterized by excellent stability and low toxicity compared to traditional emulsions [122] and could be carriers of essential oils [124] The addition of Pickering emulsion reduces the microstructural uniformity of the films and, consequently, negatively affects the structural and mechanical properties and vapor permeability. It can be solved by crosslinking [125,126].

Micro-, nanoemulsions and Pickering emulsions demonstrated different effects on the film’s properties. Thus, addition of microemulsion of cinnamon bark and nanoemulsion of thyme (wild and domestic) increased the thickness of films [94,127]. The distribution of film components is different and could lead to both roughness or smoothness [128,129,130]. Mostly, moisture content (MC), water solubility (WS), TS, elastic modulus (EM) and WVP were reduced, while changes in EAB varied. Properties of films depended on the influence of hydrophilic and hydrophobic compounds [94]. However, the elevation of TS and EAB has also been observed. In a study of nanoemulsion based on rutin introduction into pork gelatin, it is assumed that rutin can act as a crosslinking agent of the film [131]. Barrier and mechanical properties of whey protein isolate (WPI) film were improved by *Grammosciadium ptrocarpum* Bioss. nanoemulsion [130].

Release control, reduction in light transmission and UV resistance are advantages of emulsion implementation [129,131,132].

Antioxidant properties were demonstrated based on ABTS, DPPH and FRAP results, as well as antimicrobial activity against *S. aureus*, *E. coli*, *L. monocytogenes*, *S. enterica*, etc., and reduction in CFU was also noted.

### 4.3. Extracts

Phenolic compounds are the main components of plant extracts that provide both antioxidant properties and antimicrobial activity [133,134,135,136]. A number of factors affect their extraction from plant raw materials, such as solvent, selected method, duration and temperature of extraction and the ratio of extractant: solvent, particle size and affinity to solvent [137,138,139]. Water, acetone, ethyl acetate, alcohols (methanol, ethanol, propanol), etc., and their mixtures are the most popular solvents [140]. Purification of the crude extract is an important step and includes solid-phase extraction (the most popular method), matrix dispersion solid-phase extraction (allows eluating one or more classes and fractions of compounds) and liquid–solid extraction [141]. Classical extraction methods such as conventional extraction (maceration), the Soxlet method, steam- and hydrodistillation are inexpensive but time-consuming processes with low selectivity and high consumption of solvents and energy [142]. Modern effective extraction methods have been developed, including extraction with ultrasound, microwave, high pressure, high voltage electrical discharges, impulsive electric fields and supercritical fluids, taking into account profitability, energy conservation and environmental friendliness, as well as applicability on an industrial scale [143]. New methods increase the environmental friendliness of extraction by reducing the use of aggressive organic solvents, reduction processing and purification steps and increasing the yield of more thermolabile compounds [138].

Extracts from rosemary, oregano, green tea, cloves, curcumin, oregano, cinnamon, ginger, thyme and citrus fruits (for example, lemon, orange and grapefruit) are the most well studied; among them, oregano and thyme extracts were classified as the most active, while citrus extracts could effectively inhibit the growth of microorganisms [144]. The main bioactivity of a plant extract is the antioxidative effect as confirmed by elevation of DPPH, TEAC, etc., values; TBARS, myoglobin oxidation, etc.; and reduction. Several films with plant extracts affected the population of total aerobic counts, yeasts, molds and lactic acid bacteria and demonstrated antimicrobial activity against *L. innocua* ATCC33090, *S. aureus* and *E. coli*.

Distribution and affinity of the active component to the biopolymer affect the surface of the film. There are films with both a rough surface and a smooth one [145,146,147]. Addition of plant extract reduced MC and WS [148,149]. Swelling index (SI) could be elevated or reduced, depending on the added plant component, which may act as a crosslinker [147,149]. A trend of the majority of the incorporated active ingredients is the reduction in TS and elevation of EAB [150]. The combination of chitosan and quinoa protein extract enabled the elimination of plasticizers in the film production process. The resulting film exhibited a significant increase in EAB, while its water barrier properties indicated higher hydrophilicity compared to a chitosan film [151]. In few cases, an increase in TS and a decrease or slight change in EAB may be noted in the formation of starch and gelatin films, where hydrogen bonds between the active component (phenolic compounds) and biopolymer molecules can be formed [146,152]. There were no significant changes in WVP, however, in the study on films from soy concentrate and red pomegranate extract. The WVP decreased since polyphenols bind to the polar groups of protein chains, and they become inaccessible to moisture binding [148]. Addition of plant extract also affects optical properties and transparency of films [147,149].

### 4.4. Individual Compounds

The active compounds of plants can be used individually [153] and can be isolated by various methods [153,154]. Biologically active components such as thymol, carvacrol, geraniol, terpylenol and eugenol are used in many packaging materials [155]. Rosemary contains carnosine, carnosol, rosmanol, rosemadial, 1,2-methoxycarnose acid, epi- and iso-rosmanol, carnosic acid, rosmarinic acid, caffeic acid, etc. [156], while carnosic acid is the main compound [157]. Oregano contains phenolic acids and glycosides [109]. Thyme is a rich source of polyphenols, especially its leaves: caffeic acid and its oligomers, flavones, flavanones, flavanols, glycosides, etc. [158,159,160]. Cinnamon belongs to the laurel family and has a pronounced biological activity attributed to cinnamaldehyde [161]. Curcuminoid pigments are phenolic compounds, including curcumin, demethoxycurcumin and bisdemethoxycurcumin [145]. Tea polyphenols contain catechins, mono- and digallates, theaflavin, flavones, tannins, anthocyanins and phenolic acid and demonstrate high antioxidant and antimicrobial properties [162,163,164]. Tannins can be obtained from wood, leaves, stems and seeds [165]. Cocoa beans and green algae are good sources of procyanidins, catechins and its compounds [166,167]. Ginger has good antioxidant activity and contains gingerol, gingediol, gingerdion and other compounds [168]. Garlic and shallots contain two main classes of antioxidants: flavonoids and sulfur-containing compounds (allyl cysteine, diallyl sulfide and allyl trisulfide) [109]. Citrus fruits are an important source of flavonoids (hesperidin, narirutin, naringin and eriocitrin) and vitamin C [169]. Superfoods such as açaí, flaxseed, quinoa, chia seeds, pomegranate, mangosteen and blueberries are rich sources of bioactive compounds [170]. These products are notably rich in antioxidants, monounsaturated fats, fiber, polysaccharides, phytosterols and vitamins [171].

The diverse impact of plant additives on the changes of film properties is demonstrated in Table 1.

Individual plant compounds are actively used to improve the physico-chemical, structural–mechanical, barrier and organoleptic properties of biopolymer films as well as enrich them in active properties—antimicrobial and antioxidant (Table 1). The film thickness increased with the addition of individual plant compounds, which is explained by the higher content of solids in the packaging material [165,187,189]. Interestingly, addition of monolaurin and eugenol to zein film led to the reduction in thickness due to the surfactive properties of monolaurin and good distribution in the FFS. Water contact angle (WCA) was decreased, and that may be related with partial moving of added substances to the surface, which affected the wetting [186]. WCA was also reduced in the collagen films with curcumin [189]. The surface and structure of the films were different depending on the binding or crosslinking and distribution of the active components. Some films had cracks and voids [165,182,183], while others were smooth and even [186,188]. MC was reduced in active films [165,188]. The values of WS, SI and WVP varied and depended on the nature of the introduced individual plant compounds and their affinity to biopolymer. Thus, pectin films with apple polyphenols demonstrated an increase in WS and IS, which was associated with the presence of hydrophilic groups in polyphenols. The observed decrease in WVP could correspond to the reduction in the mobility of pectin chains that decreased water vapor passage through the film [184]. Sodium alginate films with thymol showed a decrease in WVP, WS and IS due to a two-stage crosslinking of the film with calcium chloride, and crosslinking of caseinate with tannins or collagen with phenolic acids led to the reduction in WVP, WS and IS [165,183,188]. The addition of carvacrol in soy protein isolate (SPI) films caused an increase in WVP, presumably due to a decrease in the availability of protein–water interaction; while in starch films, WVP increased slightly [182,187]. The values of TS and Young’s modulus (YM) decreased with addition of individual plant compounds in films, while EAB increased [186,187]. However, crosslinking or strong intermolecular interactions between the polymer and the additive without aggregation of the active compound led to an increase in TS and a slight change in EAB [183,184,189]. Weak hydrogen bonds and hydrophobic interactions between collagen and phenolic compounds insufficiently improve TS and EAB [188]. The addition of plant compounds affects the color of the films, decreases transparency and elevates the barrier properties against UV and visible spectra [182,183,187].

Plant active compounds often showed both antioxidant and antimicrobial activity (Table 1). Antioxidative effect confirmed by elevation of DPPH and reduction in TBARS, and other changes in parameters, corresponded to the confirmation of antioxidant stability. Antimicrobial activity was revealed against *S. aureus*, *E. coli*, *L. monocytogenes*, *L. innocua*, *L. grayi*, *C. albicans* and *A. niger* [165,181,183,184,185,186,187,188].

### 4.5. Waste and By-Products

Peel, pulp, husk, seeds, bark, cake, pomace, etc., are available and constitute about 30–50% of the total plant weight. The waste and by-products contain biologically active substances, including phenolic compounds, flavonoids, anthocyanins, polyphenols, tannins, etc., which may contribute to the antioxidant and antimicrobial potential of the packaging. Taking into account economic issues, increasing the waste implementation and reducing the impact on the environment are quite attractive [198,199]. Waste processing to obtain active compounds is carried out by classical extraction using water and organic solvents and their mixtures [200].

Tomatoes are a widespread crop. Tomato pomace consists of seeds, pulp and skin and is often used as feed. It is a rich source of nutrients and biologically active compounds such as carotenoids, sugars and fibers [201,202]. Grape seed extracts are rich in flavonoids (catechin and epicatechin, epicatechin-3-O-gallate, procyanidins). Naringenin and hesperidin, ascorbic acid and various organic acids also demonstrate antimicrobial properties. Red grapes are rich in anthocyanins, whereas phenolic compounds dominate in white grapes [203,204,205,206]. Grape pomace, comprising the peel and seeds, is a by-product of wine production and is notably rich in antioxidants, including quercetin and its derivatives [207]. The peel and seeds of the pomegranate are an agricultural waste. The inedible part amounts to about 50% of the total fruit weight and is a rich source of phenolic compounds, tannins, anthocyanins and flavonoids [194,206,208]. Grapefruit seed extract contains a large number of polyphenolic compounds such as flavonoids, catechins, epicatechin, procyanidins and organic acids (citric, ascorbic), which have antioxidant and antimicrobial activity [209,210]. The olive cake is one of the by-products that is generated when processing olive oil and can be a source of pectin, phenolic compounds, carotenoids and other compounds [190,211]. Olive mill wastes serve as a source of various bioactive compounds with antioxidant and antimicrobial properties. These wastes contain nutrients, anthocyanins, flavonoids, polysaccharides and phenolic compounds [212]. Onion husk represents the main waste component in onion processing (up to 60%) and contains a large number of biologically active phenolic compounds, flavonoids, etc. Purple onion husks contain quercetin and anthocyanins, while yellow ones contain more quercetin and its glycosides [191,213,214,215,216]. By-products produced during corn harvest (straw, stems, leaves, ears and husks) are a good source of dietary fiber, functional oligosaccharides and phytochemicals. Corn husk has a high cellulose content (30–50%); therefore, its derivatives, such as nanocellulose, can be obtained from it [192,217,218]. Licorice is widely used as a natural sweetener in the food industry. The main active components of licorice are glycyrrhizin and its derivatives isolated by aqueous extraction. During the extraction process, waste rich in flavonoids is often discarded [196,219]. The nut processing industry produces a large number of by-products (shells, peels, etc.) rich in phenolic compounds [193]. Watermelon seeds are a potential source of polyphenols, saponins, alkaloids and flavonoids [195].

Plant waste and by-products can be used for the production of polymer films or as a source of active components of biopolymer films (Table 1). Changes in the mechanical, physical and barrier properties of the films with plant waste are observed mainly due to the interaction of biopolymer with active plant components [191,192]. Their addition to films led to an increase in thickness [190,191,197]. It was reported that the density of the films decreased, probably due to the better particle distribution in the film matrix. Channels and cavities correlated with an increase in corn husk fiber [192]. Hydrogen bonds between the SPI and the waste extract probably led to the formation of small pores and the roughness on the surface of the film [196]. WS and MC are also reduced with addition of plant waste [191,193,197]. An increase in SI and MC of whey protein with melanin films is associated with a greater availability of hydroxyl groups due to the interaction of the main components [195]. Elevation in WVP of sodium alginate film with red onion husk extract and gelatin film with tomato pomace oil extract could be linked with an increase in the free volume of the matrix [191,197]. Films with a homogeneous structure and good dispersion of the components, on the contrary, had a significant decrease in WVP. Changes in the film structure could lead to WVP variability [190,192,196]. Increase in TS and slight changes in EAB could be linked with the formation of a dense intermolecular structure between chitosan and microparticles of olive waste, and with an increase in concentration, the structure was destabilized [190]. In low methyl pectin with corn husk fibers at ratio 5.0 g/100 g of pectin, an increase in TS was also noted [192]. The films based on whey proteins had strong hydrogen bonds between melanin and the polymer matrix, which also affected the reduction in the water vapor transmission rate (WVTR) and WCA [195]. Gelatin films with licorice residue extract also had a stable structure with a strong interaction between flavonoids and protein molecules [196]. The addition of pecan nutshell or hazelnut skin extracts partially improved the resistance to water by decreasing the solubility and increasing the WCA, improving the UV light-blocking properties in starch films [193]. The addition of plant waste and by-products affects the color of the films, decreases transparency and elevates the barrier properties against UV and visible spectra [190,191,192,193,195,196,197].

Plant waste and by-products mainly contain phenolic compounds, which are more effective against oxidative processes, confirmed by elevation of DPPH, FRAP, ABTS, radical scavenging activity, etc., of films [190,191,192,193,195,196,197,220]. Biopolymer films with by-products have great potential, as issues of the waste disposal and improvement of the characteristics of film materials are being resolved.

## 5. Microbial Biologically Active Substances

Microbial antimicrobial substances can act as natural preservatives preventing or minimizing microbiological spoilage of food products [221]. Various Gram-positive and Gram-negative bacteria produce bacteriocins [222]. Bacteriocins are low-molecular-weight (rarely more than 10 kDa) thermally stable active peptides that exhibit pronounced antimicrobial activity against various types of microorganisms [223]. According to APD3, among 3146 natural antimicrobial peptides, 383 of them are bacteriocins [224]. Bacteriocins are usually named depending on the genus of producing strain. Thus, lacticin and nisin are produced by *Lactococcus* spp., enterocin by *Enterococcus* spp., pediocin by *Pediococcus* spp., leucocin by *Leuconostoc* spp., etc. [225].

Among Gram-positive microorganisms, lactic acid bacteria (LAB) attract special attention and represent a diverse and health-promoting group of bacteria [226]. LAB have been used for canning and fermenting of various food products [227,228]. The use of LAB strains and/or their metabolites as bioconservants contributes to the effective suppression of bacterial growth [229]. Nisin, pediocin, lacticin, enterocin and bacteriocin-like inhibitory substances (BLISs) are LAB metabolites [229]. LAB and their metabolites are of particular importance and classified as GRAS (generally recognized as safe) [225]. LAB bacteriocins are generally widely used as food preservatives and exhibit inhibitory activity against closely related and unrelated microorganisms [230].

Bacteriocins possess antimicrobial activity against pathogenic and spoilage bacteria, which justifies their biotechnological potential. Broad-spectrum bacteriocins with ability to inhibit the growth of pathogenic microorganisms belonging to another genus, such as *Listeria monocytogenes*, *Staphylococcus aureus*, *Bacillus cereus,* etc., are of great interest [231]. Bacteriocins often have synergies with other treatments and can be used as components of hurdle technologies to extend food shelf life [228]. The particular interest of bacteriocin implementation in various food industries is explained by their lack of odor and color and, therefore, not affecting the organoleptic characteristics of food products [232]. In addition, bacteriocins can be easily cleaved by proteolytic enzymes such as trypsin and pepsin, which makes them safe for human consumption [230]. Based on chemical structure, molecular weight, biochemical properties, spectrum of antimicrobial activity and mechanism of antimicrobial action, bacteriocins are divided into three main classes: heat-stable bacteriocins containing lanthionine (class I); small, heat-stable bacteriocins not containing lanthionine (class II); and large heal-labile antimicrobial proteins (class III) [226].

Class I bacteriocins include several groups: lantibiotics, lipolantins, thiopeptides, botromycins, linear azole-containing peptides, sactibiotics (sactipeptides), lasso peptides, cyclic bacteriocins with a «head-to-tail» connection and glycocins [233]. Lantibiotics are the most studied group. Lantibiotics are post-translationally modified low molecular weight antimicrobial peptides (less than 5 kDa) containing lanthionine and/or methyl-lanthionine residues [234]. Based on the molecular structure, three types of lantibiotics can be distinguished—AI, AII and B corresponding to bacteriocins of linear, combined and globular conformation. AI-type lantibiotics include nisin, epilancin 15X and microbisporicin [233]. Nisin is the most popular bacteriocin with GRAS status and approved for use as a food additive. Nisin has been widely used as a food preservative for more than sixty years [12]. Nisin is a thermally stable polypeptide with molecular weight of 2–4 kDa produced by certain *Lactococcus lactis* strains and exhibited antimicrobial activity against a wide range of Gram-positive microorganisms, including *Enterococcus*, *Staphylococcus*, *B. cereus*, *Lactobacillus*, *Leuconostoc*, *L. monocytogenes*, *C. botulinum*, *C. sporogenes*, *Micrococcus* and *Pediococcus*. However, nisin demonstrates a weak inhibitory effect or its complete absence against Gram-negative bacteria, yeast and molds; therefore, it is advisable to use it with preservatives based on sorbic acid [235,236]. The low effectiveness of nisin against Gram-negative microorganisms is associated with the inability of nisin to penetrate the outer lipopolysaccharide (LPS) membrane of the bacterial cell [237]. At the same time, a number of studies have shown that nisin is an inhibitor of both vegetative cells and spores [238,239]. Since nisin is non-toxic, thermally stable and odorless, it is commercially used as preservative in various food products, including dairy and meat products, eggs, vegetables, fish, beverages and cereal-based products [240,241].

Class II bacteriocins are heat-stable, lanthionine-free peptides with a molecular weight of less than 10 kDa and divided into three subclasses—IIa, IIb and IIc [231]. Class IIa includes pediocin, sakacins, leucocin, carnobacteriocins, etc., and are called pediocin-like peptides with conserved N-terminal sequence Tyr–Gly–Asn–Gly–Val [228]. This subclass is of great interest due to its high antimicrobial activity against *Listeria monocytogenes* [242]. Pediocin PA-1 is the most widely studied bacteriocin of subclass IIa [243] and produced by *Pediococcus* spp. [242]. Pediocin is a cationic molecule with a low molecular weight (2.7–10.0 kDa) [244] with a wide spectrum of action, especially against *L. monocytogenes*, and is used as a food preservative [243,245]. Pediocin can be produced by several bacteria of this genus, the main of which are *Pediococcus pentosaceus* and *Pediococcus acidilactici* [242]. It also was reported that pediocin could be also produced by *P. damnosus*, *P. cellicola*, *P. parvulus*, *P. stilesii*, *P. Inopinatus*, *P. claussenii* and *P. Ethanolidurans* [244]. Pediocin has thermal stability and also retains activity in a wide range of temperatures and pH [246]. Enterocin is produced by *Enterococcus faecium* and demonstrates high antimicrobial activity [245]. *Enterococcus faecalis* (strains L2B21K3 и L3A21K6) produce bacteriocins with high antimicrobial activity against *L. monocytogenes* [247,248]. Cultivation media with enterocin remain active against L. monocytogenes for 90 days [247]. Strain *E. avium* DSMZ17511 demonstrated high antimicrobial activity against *L. monocytogenes* [249].

Class III bacteriocins are heat-labile proteins with high molecular weight (>30 kDa), complex activity and protein structure [231]. Megacins (from *Bacillus megaterium*), klebicin (from *Klebsiella pneumonia*), helveticin I (from *Lactobacillus helveticus*) and enterolysin (from *Enterococcus faecalis*) are members of this class [230].

The studies on the use of bacteriocins as antimicrobial agents included in the biodegradable films have been published, especially concerning nisin with GRAS status and a well-studied pediocin. The inclusion of bacteriocins in the packaging film has an advantage over dipping or spraying, which lead to the reduction in antimicrobial activity as a result of reaction with food ingredients or a decrease in their concentration due to migration into food products [12].

Films with nisin and pediocin demonstrated an inhibitory effect against pathogenic microorganisms *L. innocua*, *L. monocytogenes*, etc. [250,251,252,253]. However, some authors reported that an increase in the concentration of nisin did not lead to an increase in antimicrobial activity against microorganisms [254,255]. There was no inhibitory effect of nisin against Gram-negative bacteria, such as *E. coli*, *S. typhimurium* and *P. aeruginosa* [254,256,257]. A decrease in TS and YM in tapioca starch films was seen probably caused by a change in the structural modification of the starch in the presence of nisin. Antimicrobials may disrupt the association of biopolymer chains, reducing the number of inter-chain hydrogen bonds. High WVP is associated with the hydrophilic nature of polysaccharides used in films [258]. In some cases, EAB was elevated in gelatin films with nisin that could be explained by the presence of sodium chloride (NaCl), nisin filler, which may reduce the electrostatic repulsion between molecules and lead to a denser microstructure of the film matrix [255]. Improved mechanical properties of starch–chitosan films with the inclusion of nisin could be linked with transverse bonds formed between nisin and chitosan [259]. The addition of pediocin in a moderate concentration contributed to changes in mechanical properties of cellulose films, which indicates the possible interaction of pediocin with the cellulose matrix, which has become more rigid [252,253].

The introduction of bacteriocins into edible films leads to moderate changes in their mechanical and barrier characteristics and elicits a pronounced antimicrobial effect (Table 2). Changes in mechanical properties may be related to the interaction between chains of antimicrobial agents that can easily penetrate the film matrix.

## 6. Animal Biologically Active Substances

Active packaging with antimicrobial properties is one of the most promising [263]. Antimicrobial packaging can be prepared by incorporating synthetic or natural antimicrobial agents into films or by direct coating. Currently, plant and microbial substances with antimicrobial properties are most widely used as active components for inclusion in films [264,265,266,267]. Animal antimicrobial substances have been less studied. However, they have pronounced antimicrobial potential [268] and may become good candidates for active packaging. Many of animal antimicrobial compounds belong to enzymes, glycoproteins and antimicrobial peptides [268,269].

### 6.1. Enzymes

Antimicrobial enzymes are widespread in nature and play a crucial role in protecting organisms from a bacterial attack [270]. They have the ability to directly attack microorganisms, inhibit biofilm formation, destroy biofilm and/or catalyze reactions that lead to the formation of antimicrobial compounds [271]. Antimicrobial enzymes are used in certain technologies. For example, liquids with antimicrobial enzymes are used to clean surfaces [272]. Enzymes can be incorporated into polymer materials or cover them to prevent microbial colonization. The compositions may contain one or more enzymes or enzymes in combination with other antimicrobial agents [271].

Lysozyme was the first enzyme whose primary amino acid sequence was determined and whose structure was determined using X-ray crystallography [273]. Although lysozyme is traditionally associated with bird eggs, especially domestic chickens, it is widespread in nature and found in many sources, including some vegetables, insects, plants and fungi [274,275,276]. Lysozyme was detected in human colostrum [277], mammalian milk, saliva, mucus, blood, tears [21], macrophages, leukocytes, monocytes and neutrophilic granulocytes [273]. Lysozyme plays an important role in immune response to infections and inflammation [278]. Lysozyme derived from chicken egg protein (EC 3.2.1.17) has a bacteriostatic and bactericidal activity, mainly against Gram-positive bacteria. The mechanism of action is the cleavage of the β-1,4 bond between N-acetylmuramic acid and N-acetylglucosamine peptidoglycan chains of the bacterial cell wall [279]. Lysozyme has no activity against Gram-negative bacteria due to the presence of an outer membrane surrounding peptidoglycan chains [280]. However, Gram-negative bacteria can become sensitive to lysozyme in the presence of detergents and chelators such as ethylenediaminetetraacetic acid (EDTA), which can destabilize the outer protective membranes, making peptidoglycan molecules available for the action of the enzyme [279]. Lysozyme also exhibits antioxidant activity [281]. Lysozyme-C derived from chicken eggs was legalized as a food enzyme for use in food preservation [273]. Lysozyme is actively used to control the growth of microorganisms in foods such as cheese and wine and can be used as a preservative in other food systems [282]. Lysozyme serves as a good model of an ideal food preservative in many ways: it is an innate component of the human immune system and, therefore, should have low toxicity [277,282]; acts catalytically and can be used in low concentrations in food [283]; specific to bacterial peptidoglycan, does not react with human tissues, and also has certain properties of resistance to heat and low pH, etc. [284]. Lysozyme use in food packaging materials can extend the shelf life of non-sterile or minimally processed foods by preventing a microorganism’s growth [284]. It has been shown that poly-(l-glutamic acid) nanofilms with egg lysozyme inhibit the *Microcccocus luteus* growth [285]. Edible pectin antimicrobial film technology capable of controlling the release of lysozymes is studied. The presence of pectinases enhances the release of lysozymes, which confirms the expediency of using the developed edible antimicrobial film to protect food from lysozyme-sensitive microorganisms, especially those that produce pectinolytic enzymes [279]. Lysozyme addition to the film-forming solution contributed to the antimicrobial activity of films and affected the mechanical properties. Thus, lysozyme addition to low methoxyl (LM) pectin led to an increase in YM and a slight decrease in EAB [279], while addition to jackfruit seed starch strongly affected YM, EAB, TS and WVP [280] in a dose-dependent manner.

Lactoperoxidase (LP) is a hemoprotein presented in milk, tears and saliva [286] and is the most common enzyme in cow’s milk [287]. The peroxidase activity associated with cow’s milk was first demonstrated by Arnold in 1881, and a protein called lactoperoxidase was isolated by Theorell and Akeson in 1943 [273]. The interaction of lactoperoxidase-thiocyanate-hydrogen peroxide forms the so-called lactoperoxidase system (LPS), in which hydrogen peroxide serves as a substrate for LP during the oxidation of thiocyanate (SCN−) and iodide ions, which leads to the formation of highly reactive oxidants [286]. The association of lactoperoxidase with microbial growth inhibition was first demonstrated by Wright and Trammer (1958) [288], whereas the characterization of the entire LP system, including enzymes and substrates, was carried out later [289]. In addition, the LP system has hexokinase and glyceraldehyde-3-phosphate dehydrogenase activities [290], which can contribute to the antimicrobial action of the system [273]. The LP system has the ability to suppress bacteria, fungi, parasites and viruses and is thus considered a natural broad-spectrum antimicrobial agent [291]. It has been shown that during pasteurization, milk loses about 75% of LP activity, while purified LP becomes unstable after 15 min [273]. Thermal denaturation of LP in milk, whey, permeate and buffer begins at about 70 °C, and the concentration of calcium ions affects the thermal sensitivity of LP [292]. The thermal stability of lactoperoxidase is lower in an acidic environment (pH 5.3) and may be associated with the release of calcium from the molecule [293]. LP is deactivated during storage at pH 3 with partial denaturation at pH < 4, while at pH values up to 10, enzyme deactivation does not occur [293,294]. The adsorption of LP on certain surfaces can cause a significant decrease in activity [273]. The use of the LP system as a natural preservative in food products, in particular in dairy, has increased significantly after the introduction of industrial processes for the isolation of LP from milk and whey [293]. The LP system’s addition to biodegradable films enhanced antimicrobial activity and did not significantly affect barrier properties, but can affect the mechanical ones. Thus, LPS with combination with chitosan extended the shelf life of cooled fish [295], while incorporation in defatted soybean meal-based (DSM) films led to antimicrobial activity against *S. typhimurium* [296]. Shokri et al. investigated the effectiveness of the LP system in combination with whey protein to create edible food coatings, and the shelf life was increased to at least 16 days in the presence of LP system [297]. The enzyme LP may be a good candidate for the inclusion into biopolymer films and coatings to extend the shelf life of food products [294,295,296,297,298,299,300].

### 6.2. Glycoproteins

Glycoproteins are proteins in which carbohydrates (glycans) are covalently bonded to proteins [301]. The proportion of glycans ranges from two to thirty percent or ranges from fifty to sixty percent or more of the total mass of the molecule [302]. These molecules have many properties such as biodegradability, biocompatibility, non-toxicity, antimicrobial and adsorption properties; therefore, they have a wide range of applications.

Lactoferrin (LF) is also called lactotransferrin or lactosiderophilin [268]. LF is an iron-binding bioactive glycoprotein of the transferrin family, which contributes to the control of iron in biological fluids. LF is found mainly in milk, on the surface of mucous membranes (for example, in intestinal epithelial cells) and mammalian exocrine secretions such as saliva, tears and seminal fluid, as well as in secondary granules (vesicles) of polymorphonuclear neutrophils or lymphocytes [303,304]. Human and pig’s milk contain significantly higher concentrations of lactoferrin (almost ten times) than cow’s milk [273,305]. Nevertheless, the highest concentrations of LF are present in the colostrum of cattle; at the same time, an increase can also be observed in milk after a mastitis infection [273].

LF plays an important role in many physiological mechanisms, such as the adsorption of metal ions in the intestinal tract [306], promoting digestion and assimilation of micronutrients and macronutrients from milk [307], suppression of myelopoiesis [308], protection of the intestinal flora of young animals from enteropathogenic bacteria [309], protection from mastitis [310], immunoregulatory function (i.e., contribution to the pre-immune innate protection of mammals) and opsonic activity [273]. LF has antioxidant properties and demonstrates a wide antimicrobial spectrum, including antibacterial, antifungal, antiprotozoal, antiviral and antitumor properties [268,311], and LF addition in films could affect barrier and mechanical properties (Table 3). 

Bacterial cellulose films with bovine LF significantly inhibit *E. coli* and *S. aureus* growth, but are characterized by a decrease in mechanical properties [313]. Combination of LF and lysozyme in cellulose-based food packaging [316] demonstrated wider antimicrobial activity.

Ovotransferrin (OTF, also called conalbumin) is an iron-binding monomeric glycoprotein that makes up at least 10–12% of the total solids of egg whites [319]. OTF isolation and purification can be performed using solvent fractionation and chromatographic methods (ion exchange chromatography or metal affinity chromatography) [320]. OTF has a high affinity for iron [321]; therefore, stoichiometric balance of iron affects the OTF [273]. Saturation of OTF with iron reduces its effectiveness against many Gram-negative bacteria [322]. However, OTF remains effective against Gram-positive bacteria, including lysozyme-resistant strains, at 30–39.5 °C, regardless of the presence or absence of iron [323]. Alkaline pH and elevated temperature (~40 °C) enhance the antimicrobial activity of OTF [323]. On the other hand, OTF is thermosensitive, and 80% of the activity is lost when heated to 70–79 °C for 3 min or 60 °C for 5 min [324]. OTF is considered to have mainly a bacteriostatic activity, although there is evidence of a biocidal effect against a wide range of bacteria such as *E. coli*, *Klebsiella* spp., *Proteus* spp., *Pseudomonas* spp. and *S. aureus* [273]. It is reported that OTF prolongs the lag-phase and reduces the growth rate of many Gram-positive and less sensitive Gram-negative microorganisms [320]. Among Gram-positive bacteria, *Bacillus* spp. and micrococci are the most sensitive to OTF [273]. An inhibitory effect has also been observed against *Candida* spp. [325]. Despite the negative effect of iron saturation on the OTF antimicrobial activity, it is reported that the complex of OTF with other metal cations increases its antimicrobial effectiveness [326]. Biopolymeric film made of κ-carrageenan with OTF could extend the shelf life of chilled chicken breast (Table 3); however, the addition of EDTA was recommended to enhance the antimicrobial effect [314]. OTF addition to gelatin demonstrated poor mechanical characteristics, but with pronounced antimicrobial activity against *E. coli*, *F. psychrophilum*, *S. putrefaciens* and *P. florescens* [315].

Avidin is another glycoprotein isolated from the protein of various bird eggs. Avidin is a positively charged glycoprotein with a mass of 66 kDa and can also be found in egg jelly of invertebrates [327]. Discovery of the antibacterial molecule streptavidin produced by *Streptomyces* spp. (avidin analog 60 kDa) showed a similar primary structure compared to avidin and confirmed suspicions that avidin has antimicrobial properties [273]. Although the antimicrobial activity of avidin has not been established, it has been suggested that the compound is involved in antimicrobial responses based on streptavidin production by *Streptomyces* during the formation of an antibiotic system and supported by the initiation of avidin production at a site of injured tissue in chickens [328]. It is assumed that the production of avidin and its secretion by macrophages are induced during inflammation and cellular damage and as such may constitute a host protection factor against bacterial and viral infection [329]. Miller and Tausig (1964) showed an increased amount of avidin in chicken tissues after intraperitoneal and intravenous administration of *E. coli* that confirmed the opinion that avidin is aimed at combating microbial infection [273,330]. It has been hypothesized that due to the high affinity of avidin for biotin, it can act as an antimicrobial agent, making biotin inaccessible to microorganisms that need it [331]. It has been shown that avidin inhibits in vitro yeast and bacterial growth [327].

### 6.3. Histones and Antimicrobial Peptides as Potential Agents for Inclusion into Biopolymer Matrices

Histones or histone-derived fragments have antimicrobial activity in vertebrates, from fish to humans. The antimicrobial activity of histones was first demonstrated in 1958 for histones A and B purified from calf thymus and exhibited activity against various Gram-positive and Gram-negative bacteria [332].

Fish antimicrobial histone proteins have been found in skin mucus or liver tissue: H2B-like proteins in catfish skin [333], H2A in trout skin [334] and H1 in Atlantic salmon liver [335]. Pat et al. found high levels of histones H2A, H2B, H3 and H4 in the hemocytes of the Pacific white shrimp *Litopenaeus vannamei* and demonstrated their activity against *Micrococcus luteus* [332].

A mixture of histones (H1, H2A, H2B, H3, H4 and H5) extracted and purified from chicken erythrocytes has antimicrobial activity against various Gram-negative and Gram-positive planktonic bacteria and Gram-positive bacterial biofilms [336]. Jodoin and Hincke reported that histone H5 isolated from chicken erythrocytes has a powerful broad-spectrum antimicrobial effect against Gram-positive and Gram-negative planktonic bacteria (MIC range: from 1.9 ± 1.8 to 4.9 ± 1.5 micrograms/mL), including vancomycin-resistant *Enterococcus* and methicillin-resistant *S. aureus* and anti-biofilm activity against *L. monocytogenes* и *P. aeruginosa* biofilms [337]. Histones can be isolated from chromatin by acid extraction [338]. Due to the pronounced antimicrobial activity, histones could be attractive as antimicrobial agents in active food packaging. Nevertheless, the issues of their use as active components of food packaging need to be experimentally confirmed.

Antimicrobial peptides (AMPs) are identified in almost all species, from bacteria to humans, and have a wide range of antimicrobial activity against bacteria, fungi, viruses and eukaryotic parasites [339,340]. AMPs form an important part of the “innate” resistance of the host organism, acting as the first line of defense against infection. It is important to note that AMPs are considered to have a completely different mechanism of action from clinically used antimicrobial agents [341]. The main mechanisms responsible for the antimicrobial activity of AMPs are associated with the permeability of target membranes and the subsequent leakage of cells, inhibition of RNA, DNA and protein synthesis as well as a decrease in cell viability [289]. There are studies on the inclusion of bacterial and plant AMPs into biopolymer films for active packaging [245,342], but few studies concerning animal ones.

According to APD3, among 2463 AMPs from animals, 373 from mammals, 619 from arthropods and 146 from fish were annotated [224]. Animal AMPs are numerous and include such classes as defensins, cathelicidins, hepcidins, histatins and lactoferricins (derived from lactoferrin) [343]. Defensins and cathelicidins are one of the most widely expressed in mammals [344,345]. Synthesis of recombinant AMPs with a known or bioinformatically calculated sequence for their further use is quite popular compared to their extraction from native animal tissues. These technologies are in high demand, but obtaining the recombinant mammalian AMP presents some difficulties. The studies on new AMPs are more widespread than the investigation of the effective AMP isolation, e.g., from insects [346] and invertebrates [347]. The issues of native peptides’ extraction, in particular AMPs, are quite complex, while isolation and practical application of protein hydrolysates are more studied. Protein hydrolysates are studied as food preservatives and include porcine blood protein hydrolysate [348], gelatin hydrolysate from blacktip shark skin [349] and fish collagen hydrolysate [350]. Integration of protein hydrolysates into edible films and coatings was used to suppress the growth of microorganisms [351] (Table 3). Polypeptide fraction is used without purifying specific peptides, and the activity of these fractions can be not only antimicrobial, but also antioxidant, antifungal, etc.

The issue of using extracted and purified endogenous animal AMPs as active components of biopolymer packaging materials is not well discussed. However, some AMPs may be promising candidates for inclusion into the biopolymer matrix. Thus, pleurocidin from the skin of winter flounder *Pleuronectes americanus* [273] demonstrated activity against Gram-positive *S. aureus*, *L. alimentarius* и *L. monocytogenes* and Gram-negative *E. coli*, *S. typhimurium* и *Vibrio* spp. as well as against yeast and mold fungi. This broad-spectrum activity points to the potential of this antimicrobial peptide as a food preservative [268,352]. There are quite a lot of studies on insect AMPs. Thus, cecropins B and P1 showed elevated inhibitory activity against *E. coli*, which is found in milk, meat and vegetables [346]. Peptide Hf-1 isolated from the larvae of *Musca domestica* (Diptera:Muscidae) demonstrated bactericidal activity against *E. coli*, *P. aeruginosa*, *S. typhimurium*, *S. dysenteriae*, *S. aureus* and *B. subtilis* [353]. Jelleine-1 from royal jelly of honey bees has a strong antimicrobial activity against food-borne pathogen *L. monocytogenes* by both pore formation and effect on DNA; the formation of biofilms was also significantly reduced [354]. Persulcatusin is an antimicrobial peptide of the *Ixodes persulcatus* that showed the highest activity against methicillin-resistant *S. aureus* without visible damage to the mammalian and human cells [355]. Thus, pronounced antimicrobial activity made AMPs potential agents for the inclusion into the biopolymer matrices for the development of promising active packaging materials to slow food spoilage and increase their safety and shelf life.

## 7. Organic Nanoparticles

Various organic, inorganic and combined nanoparticles (NPs) are used in the development of effective food packaging [356]. The inorganic NPs are the most variable and include transition and alkaline earth metals, non-metals, metal oxides, nanoclay and graphene oxide and are commonly used in food packaging materials [357]. Biopolymer organic nanoparticles/nanobeads are easy to prepare and highly stabile in biological fluids and during storage [358]. Organic nanoparticles are different in types: micelles, dendrimers, liposomes, nanogels, polymeric NPs and layered biopolymer [359]. The most used organic NPs in food packaging are nanocellulose (NC), chitosan NPs and starch NPs; nanofibers, nanoplatelets, nanotubes and nanowires can also be used [357].

NC can be isolated from plants or synthesized by bacteria. NC produced from plants can be classified into two types: cellulose nanofibrils (CNFs) and cellulose nanocrystals (CNCs), while NC produced by many species of bacteria is the bacterial nanocellulose (BNC) [360,361]. NC lacks antimicrobial and bioactive properties [362]; therefore, NC is usually used as a reinforcement agent for bio-based films [363]. The most common strategy used for biopolymer composite synthesis based on BNC is in situ technique, which involves the addition of materials (e.g., sodium alginate, carboxymethylcellulose (CMC), gelatin, agar, pectin, starch) to the culture medium at the beginning of the BNC production process [364]. Chitosan can be synthesized into NPs (ChNPs) via many methods such as ionic gelation, reverse emulsion and polyelectrolyte complexation [365]. One of the most investigated properties of chitosan is its antimicrobial effect embracing from food to agriculture applications [366]. In addition, ChNPs can enhance mechanical strength of biodegradable plastics [367] (Table 4). Starch nanoparticles (StNPs) demonstrated strong reinforcing effects, a positive impact on barrier packaging and could be prepared by hydrolysis, emulsion, ultrasonication, self-assembly, nanoprecipitation, etc. [365]. Starch itself does not have antibacterial [368] properties and could demonstrate antioxidant action due to loading various phenolic compounds [369,370]. Protein-based NPs can be natural or synthesized and obtained via natural self-assembly, chemical or physical [365,371,372]. Protein-based NPs are used in food packaging to enhance the strength and barrier properties such as water barrier properties [356]. However, due to unique properties, protein-based NPs are excellent carriers of bioactive substances as a component of active packaging [373]. Encapsulation into lipid-based, polymeric-based and nanoclay-based NPs can protect and control the release of active compounds and can enhance the performance of biopolymeric matrices [374]. Therefore, organic NPs are often used not only as reinforcing agents, but as a carrier of antioxidants, antimicrobials, inorganic NPs, etc., for active packaging development [66,374,375,376,377,378].

## 8. Conclusions

Polymer packaging allows to increase the shelf life of products and to reduce or eliminate exposure to light and heat, additional contamination and excessive development of microorganisms, thereby reducing product oxidation and microbial spoilage. The growing concern about the environmental pollution associated with the widespread use of plastic packaging requires the search of alternative renewable resources that are biodegradable for the sustainable production of biopolymers as a packaging material. Biofilms are mainly based on hydrophilic polysaccharide and protein polymers, which may include lipid components to increase hydrophobicity. In addition to the edible/biodegradable packaging development, research in this area has led to the creation of active and intelligent packaging. Active packaging provides reduction in microbial, oxidative and enzymatic spoilage, possible contamination, weight loss and changes in color and integrity of products during storage. The use of active additives in the packaging provides a number of advantages compared to direct introduction into food—the use of a lower concentration of active substances, controlled release and a decrease in stages of technological processing of products. Addition of plant, microbial, animal and organic nanoparticles into bio-based films is extremely relevant due to safety and direct activity.

## Figures and Tables

**Table 1:** Application of plant additives in food biodegradable packaging.

Source	Amount Added	Biopolymer Matrix	Changes in Film Properties	Changes in Activity	Refs.
Essential Oils					
Eucalyptus	0.5–4 wt.%	chitosan/glycerol/tween 80 (1.5 g in 100 mL acetic acid 0.7% (*v*/*v*):0.15 g/g chitosan) chitosan/glycerol/tween 80 (2 g in 100 mL acetic acid 1% (*v*/*v*):0.5 mL/g chitosan)	A decrease in WS, TS, MC, homogeneity, transparency, increased WVP, EAB. Changes in color.	Antimicrobial activity against *E. coli*, *S. enterica*, *B. cereus*, *S. aureus*, *P. aeruginosa*, *C. albicans* and *parapsilosis*.	[107,108]
Lemon, thyme, cinnamon and mixture	1 mL or blend 0.5 mL + 0.5 mL	chitosan/glycerol/tween 20 (2 g in 100 mL acetic acid 1% (*v*/*v*):0.6 g:0.1 g)	The films were slightly rough. An increase in thickness, TS and glow. A decrease in WS, swelling degree (SD), WVP, WC, EAB and transparency.	Antimicrobial activity against *S. aureus* (more pronounced) and *E. coli*.	[97]
Rosemary	0.5–1.5% *w*/*v*	chitosan/tween 80 (2 g in 100 mL acetic acid 1% (*v*/*v*):0.2% (*w*/*v*))	Heterogeneous and rough film structure. A decrease in WS, TS and light transmission in UV spectra. An increase in WVP (at ≥0.5% rosemary), EAB and transparency.	Antimicrobial activity against *L. monocytogenes*, *P. putida*, *S. agalactiae*, *E. coli* and *L. Lactis*.	[95]
Garlic	Previous (10%) dilution with ethanol 0.1%, 0.2%, 0.3% and 0.4% *v*/*v*	sodium alginate/glycerol (1 g:0.4 mL)	An increase in WVP and EAB (at ≤0.4% garlic). A decrease in TS. Minor changes in color.	Antimicrobial activity against *E. coli*, *S. typhimurium*, *S. aureus* and *B. cereus*.	[172]
Oregano	0.5, 1.0 and 1.5% *w*/*v*	sodium alginate/glycerol/tween 80/CaCO_3_/glucono-δ-lactone (GDL) (1.5 g:0.243 g/g of alginate:0.250 g/g of essential oil:0.03 g/g alginate:5.4 g/g CaCO_3_)	Small pores and yellowish. An increase in thickness, EAB and WVP. A decrease in TS and transparency.	Antimicrobial activity against Gram-positive (high) *S. aureus* and *L. monocytogenes* and Gram-negative (less pronounced) *E. coli* and *S. enteritidis*.	[96]
Ginger	0.5% *w*/*w*	hydroxypropylmethylcellulose (HPMC)/tween 80 (5 g:0.25 g)	A decrease in TS, EAB and WVP. An increase in oxygen permeability (OP). Slight darkening.	Antioxidant activity: peroxide value (PV) reduction.	[173]
Clove, cinnamon and mixture	0; 0.5; 1; 1.5; 2; 2.5; 3; 3.5; and 4% (*v*/*v*)	corn starch/glycerol/xanthum gum/cooking oil (6 g:50% (*w*/*w*) of starch:0.1% (*w*/*v*):2% (*v*/*v*))	Changes in color.	Antioxidant activity: TBARS reduction. Antimicrobial activity against *L. monocytogenes*, *L. lactis* (high) and less pronounced against *L. mesenteroides*, *P. fluorescens*, *S. putrifaciens*, *E. coli* and *S. typhimurium*. MIC—1%.	[174]
Oregano and black cumin	0.5–2.0% *w*/*v*	maize starch/glycerol/guar-xanthan (1.5 mg/mL:40% (*w*/*v*) of starch:0.1% (*w*/*v*) of starch)	An increase in thickness and EAB. A decrease in TS, transparency, SD, WVP and WC. Changes in color.	Antioxidant activity: DPPH increases. Antimicrobial activity against *S. Typhimurium*, *E. coli* and *L. monocytogenes*.	[100]
Rosemary, oregano and mixture	1.82, 0.48 and 0.91 + 0.24 mg/mL	pectin/glycerol (1 g:1 mL)	The use on fresh broccoli did not show unacceptable sensory changes.	Antioxidant activity: DPPH and ABTS elevation. Antimicrobial activity against mesophilic bacteria, coliforms, yeasts and molds.	[175]
Clove	0.75 mL/g of protein	sunflower protein concentrate/glycerol (5 g:1.5 g)	Inhomogeneities on surface. A decrease in WS, transparency and EAB. Slight increase in TS and thickness. Changes in color.	Antioxidant activity: ABTS and FRAP increase, low TBARS levels. Antimicrobial activity against *A. hydrophila*, *A. niger*, *B. cereus*, *B. coagulans*, *B. animalis subespecie lactis*, *B. bifidum*, *B. thermophacta*, *C. freundii*, *C. perfringens*, *D. hansenii*, *E. faecium*, *E. coli*, *L. acidophilus*, *L. helveticus*, *L. innocua*, *L. monocytogenes*, *P. expansum*, *P. phosphoreum*, *P. aeruginosa*, *P. fluorescens*, *S. cholerasuis*, *S. putrefaciens*, *S. sonnei*, *S. aureus*, *V. parahaemolyticus* and *Y. enterocolítica*.	[80]
Oregano	0.5–1.5 wt.%	WPI/sorbitol (8 g and 5 g:3 g and 1.875 g)	An increase in EAB. A decrease in glass transition temperature (Tg), TS and YM.	Antimicrobial activity against *P. aeruginosa* and *Lactobacillus* spp. Reduction in bacterial total viability count (TVC).	[99]
Cinnamon, ginger	2.5%, 5.0%, 7.5%, 10% *w*/*w* of protein	sodium caseinate/glycerol (8 g:2.4 g)	Roughness decreased. Reduction in TS and EAB in a dose-dependent manner, correlated with humidity level. EM decreased at 10% humidity and increased at 5% humidity. An increase in WVP correlated with the level of humidity. Changes in color.	Antioxidant activity: peroxide value (PV) reduction.	[176]
Ginger, turmeric, plai	25%, 50%, 100% *w*/*w* of gelatin	fish skin gelatin/glycerol/tween 20 (3.5 g:30% (*w*/*w*) of gelatin:glycerol:25% (*w*/*w*) of essential oils)	A decrease in WVP, TS and light transmittance. An increase in thickness, hydrophobicity, EAB and yellowness.	Antioxidant activity: ABTS and DPPH increase.	[101]
Thyme	2, 4, 6, 8% (*w*/*w*)	collagen hydrolysate/glycerol/tween 80 (8 g:2 g:0.5 g)	Roughness decreased. An increase in thickness, EAB and yellowness. A decrease in TS, solubility, light transmittance and transparency.	Antioxidant activity: DPPH elevation.	[102]
Melissa officinalis	0.1, 0.15, 0.20% (*v*/*v*)	pectin/collagen/glycerol/tween 80 (1.5 g:1 g:1.25 g:0.2 g)	Less transparent, but uniform and flexible. A decrease in WVP, WC and TS. An increase in thickness and EAB. Changes in color.	Antioxidant activity: ABTS and DPPH increase.	[177]
Emulsions					
Thyme oil (microemulsion in RH40 and Span80 (2:1))	1%, 2.5%, 5%, 10%, 20% (*v*/*v*)	potato starch/glycerol (4 g:1.5 g)	Reduced the release of the active components. Slight changes in color.	A decrease in the total number of colonies. Antimicrobial activity against *S. aureus* and *E. coli*.	[132]
Cinnamon bark oil (CBO, microemulsion with soybean oil (SBO), tween 80, propylene glycol/water)	1, 2, and 3% *w*/*w* (CBO/SBO (1:0, 2:1, 4:1))	chitosan/glycerol (2 g in 100 mL acetic acid 1% (*v*/*v*):0.4 g)	A decrease in MC and TS. An increase in thickness, WVP, EAB and yellowness.	Antimicrobial activity against *L. monocytogenes*, *E. coli* and *S. enterica* (at >1% microemulsion).	[94]
Copaiba oil (CO) (nanoemulsion with tween 80)	1:1 (CO of water 1, 3, 6%:FFS)	pectin/glycerol (6 g:15% (*w*/*w* of pectin))	An increase in roughness, EAB and hydrophobicity. A decrease in thermostability, EM, TS and WVP. Changes in color. The ability to biodegrade.	Antimicrobial activity against *S. aureus* and *E. coli*.	[128]
Ginger essential oil (GEO Pickering emulsion stabilized by cellulose nanocrystals (CNCs))	3 g GEO:0.3 g CNC in 60 mL of deionized water	sodium alginate/glycerol (6 g to 240 mL of deionized water:2 g)	Smooth, dense and uniform surface without cracks. A decrease in MC, WVP, EAB and TS. An increase in YM. WCA value corresponded to hydrophilic surface. High transparency and satisfactory barrier properties in relation to UV. Slow release of the active component.	Antioxidant activity: ABTS and DPPH increase.	[129]
*Thymus daenensis* essential oil—wild and cultivated (nanoemulsion with tween 80, lecithin)	2 mL	HPMC/polyethylene glycol (16 g:1 g)	An increase in thickness. A decrease in TS, EAB and EM.	Antimicrobial and antifungal activity against *E. coli*, *S. typhi*, *S. Dysenetriae*, *S. flexneri*, *S. aureus*, *S. epidermidis*, *B. subtilis*, *E. faecalis*, *E. faecium*, *A. baumannii*, *K. pneumoniae* and *C. albicans*.	[127]
Rutin (nanoemulsion with SBO, spin 80, tween 80)	5, 10, 15 or 20% (*w*/*w* of the gelatin)	gelatin of pig skin/glycerol (6.25 g:30 g/100 g of gelatin)	A decrease in light transmission, MC and WS. An increase in hydrophobicity, WVP, EAB and TS.	Antioxidant activity: ABTS, FRAP and DPPH elevation.	[131]
*Grammosciadium pterocarpum* Bioss. essential oil (GEO nanoemulsion with tween 80 (HLB-15) and water), GEO/tween 80 (4:1)	0.5, 1 and 1.5% of WPI	WPI/glycerol (5 g:2.5 g)	Improved the integrity, absorbed less moisture. An increase in TS and EAB. Reduction in WVP.	Antioxidant activity: DPPH increases. Antimicrobial activity against Gram-negative (*S. typhimorium*, *E. coli*, *P. aeruginosa)* and Gram-positive (*L. monocytogenes*) microorganisms.	[130]
Extracts					
Green tea (ethanol)	5% (*w*/*w* of starch)	potato starch/glycerol (2.5 g:0.6 mL)	Smooth and uniform. A decrease in WVTR, EAB and WS. An increase in Tg, enthalpy transition (H) and TS.	Antioxidant activity: DPPH increases, TBARS reduction, inhibition of oxymyoglobin oxidation.	[146]
Ginseng (ethanol in water (1:8))	0.5 g/mL of FFS	sodium alginate/CaCl_2_/glycerol (2 g:0.01 g:3 g) After drying, 2% CaCl_2_ solution was poured onto the surface of films for 30 s and then dried.	An increase in EAB and transparency. A decrease in WS, TS and EM. A slight increase in WVP.	Antioxidant activity: DPPH increases.	[150]
Green tea (water)	20% (*w*/*v*)	chitosan/glycerol (2 g in 100 mL acetic acid 1% (*v*/*v*):30% (*w*/*w* of chitosan))	The cutting force increased. Changes in color. Wrapped samples of pork sausages had a more acceptable smell and color.	Antioxidant activity: TBA reduction. Antimicrobial activity: population of total aerobic counts, yeasts and molds, and lactic acid bacteria decreased.	[178]
Murta fruit (50% ethanol)	0.25 *w*/*w* of MC	methyl cellulose (MC)/glutaraldehyde/polyethylene glycol (1 g in 100 mL ethanol 70%:10, 15, 20% (*w*/*w*) of MC:25% (*w*/*w*) of MC)	Small pores and heterogeneity. A decrease in SI and EAB. An increase in thermostability, TS, EM and yellowness.	Antioxidant activity: ABTS elevation. Antimicrobial activity against *L. innocua*.	[147]
Green tea	0.1–0.2 mg/mL of FFS	whey protein concentrate/glycerol (1 g or 2 g:5 g or 8 g)	-	Antioxidant activity: DPPH increases and the β-carotene bleaching test, decrease in p-anisidine and TBARS.	[179,180]
Red grape	0–10% (*w*/*w* of protein)	soy protein concentrate/glycerol (5 g:30% or 40% (*w*/*w* of protein)) in phosphate buffer solution pH 10	An increase in EAB and EM. Reduction in MC, transparency and WVP. A slight increase in overall color difference (ΔE); 30% glycerin—significant increase in EAB; 40%—decrease in TS and EAB.	Antioxidant activity: FRAP and DPPH elevation.	[148]
Curcuma (ethanol)	5, 50, 100, 150 and 200 g/100 g of gelatin	gelatin of pork skin/sorbitol (2 g:30 g/100 g of gelatin)	Less uniform structure. An increase in TS and EAB. A decrease in WS, WVP, EM and glow. Changes in color.	Antioxidant activity: ABTS and DPPH elevation.	[145]
Berberis lyceum root (methanol)	1, 2, 4% (*w*/*v*)	collagen of fish skin/carboxymethylcellulose/glycerol (3 g:1.5 g:0.05% (*w*/*v*))	A decrease in transparency. An increase in thickness, SI, WS and WCA. High biodegradability and barrier properties against the UV–visible spectra.	Antioxidant activity: DPPH, nitric oxide scavenging, FRAP and TAC increase.	[149]
Cinnamon, guarana, rosemary and boldo do chili (ethanol)	1% (*w*/*v*)	gelatin of pork skin/chitosan or blend (75%:25%, 50%:50%) (4 g/1 g in 100 mL acetic acid 1% (*v*/*v*))	An increase in EAB, glow and yellowness. A decrease in TS and EM. Changes in color.	Antioxidant activity: TEAC elevation. Antimicrobial activity against *S. aureus* and *E. coli*.	[161]
Individual compounds					
Green tea polyphenols	30% (*w*/*w* of chitosan)	chitosan/glycerol (2 g in 100 mL acetic acid 1% (*v*/*v*):0.5% (*w*/*v*))	-	Antioxidant activity: TBARS and metmyoglobin reduction. Antimicrobial activity: the total microbial contamination decreased.	[181]
Carvacrol, linalool and thymol	2%, 4% and 6% (*w*/*w*)	starch/water/glycerol (63%, 61% or 59% (*w*/*w*):10% (*w*/*w*):25% (*w*/*w*))	Voids in the structure. A decrease in TS and light transmission. An increase in EAB and EM; WVP increased slightly.	-	[182]
Thymol (ethanol)	0.01, 0.1, 1.0, 10 mg/mL	sodium alginate/tween 80/glycerol (1 g:1 g:1 g); 2 stages of crosslinking: (1) Ca^2+^ at a concentration of 0.01 g/100 mL in film-forming solution for 1 h; (2) after drying, dipping into 1.0 L of aqueous Ca^2+^ solution [2.0 g/100 mL (*w*/*v*) Ca^2+^, 3% (*v*/*v*) glycerol] for 15 min and rinsing with glycerol solution (5%, *v*/*v*)	A decrease in WS, IS, WVP, transmission coefficients of UV and visible light. Smoother, but cracks appeared at 10 mg/mL. The highest TS was observed at 1.0 mg/mL, and EAB at 0.1 mg/mL; the lowest TS and EAB, at 10 mg/mL. Reduction in the weight loss of sliced apples and better color preservation.	Antioxidant activity: DPPH elevation. Antimicrobial activity against *S. aureus* and *E. coli*.	[183]
Polyphenols from apple	0.5%, 1% and 1.5% (*v*/*v*)	citrus pectin/glycerol (2.75 g:30% (*w*/*w*) of pectin) +30 mL CaCl_2_ solution (1% *w*/*w* of pectin)	A decrease in MC, EAB, WVP and transparency. An increase in thickness, density, WS, SI and TS. Changes in color.	Antioxidant activity: DPPH elevation. Antimicrobial activity against *S. aureus*, *E. coli* and *L. monocytogenes*.	[184]
Capsaicin	0.1, 0.3 and 0.5 g	ethyl cellulose/castor oil (2 g in ethanol 95% for 45 mL solution:0.7 mL)	An increase in EAB. A decrease in TS (at >0.1 g capsaicin) and water absorption.	Antioxidant activity. Antimicrobial activity against *S. aureus* and *E. coli*.	[185]
Tannins from white, red peel grape and oak bark	1:3 g of protein	sodium casein/glycerol (2 g:0.3 g/g of casein)	No breaks, compact with microcracks, slightly yellow and glowing. A decrease in MC, WS, EAB and WVP. An increase in thickness and EM. A slight increase in TS.	Antioxidant activity: DPPH elevation. Antimicrobial activity against *E. coli* and *L. innocua*.	[165]
Monolaurin, eugenol and mixture	3.0, 5.0/2.0, 3.0% (wt)	zein/ethanol/glycerol (powder:17% (wt):4% (wt))	Flat and plate structures. A decrease in TS and WVP; an increase in EAB. The thickness of the films varied: increased with eugenol addition, decreased with laurine addition, including when adding a mixture. WCA was reduced, but all films could be classified as hydrophobic.	Antimicrobial activity against *E. coli*, *S. aureus*, *C. albicans* and *A. niger*.	[186]
Carvacrol	1%, 2%, or 3% (*w*/*v*)	SPI/glycerol (5 g:0%, 1%, 1.5%, 2%, 2.5% or 3% *w*/*v*) 1.5% glycerol for all concentrations of carvacrol; for other glycerol, 1% carvacrol	A decrease in TS, YM and transparency. An increase in thickness, EAB and WVP. Good barrier properties against UV spectra.	Antimicrobial activity against *L. grayi* and *E. coli*.	[187]
Laccase-oxidized vanillic acid, protocatechuic acid, gallic acid, ferulic acid, caffeic acid	20 mmol/L (30 mL)	collagen sponge (5 mg/mL in 0.1 mol/L acetic acid)	Smooth and uniform surface, no visible phase separation. Low MC value and transparency. An increase in TS, thermal denaturation temperature (Td) and EAB (slight). A decrease in WVP. Barrier properties against UV and visible spectra. Changes in color.	Antioxidant activity: ABTS and DPPH elevation. Antimicrobial activity against *S. aureus* and *E. coli*.	[188]
Curcumin	0.25, 0.5, 1.0 and 1.5% (*w*/*w* of gelatin)	gelatin A/sodium dodecyl sulfate/glycerol (5 g:1% (*w*/*w* of gelatin):30% (*w*/*w* of gelatin))	Smooth surface, no visible damage, transparent with a yellow tint. A decrease in EAB (till curcumin < 1%), WVP, WCA and UV light transmittance. An increase in thickness, TS and EM.	Antioxidant activity: ABTS and DPPH elevation. Antimicrobial activity against *E. coli* and *L. monocytogenes*.	[189]
Waste and by-products					
Microparticles of olive pomace	10%, 20% and 30% of chitosan	chitosan/glycerol (2 g in 100 mL acetic acid 1% (*w*/*w*):1% (*w*/*w*))	Smooth surface with lots of drops of air and oil. No significant influence on WVP and WS. A decrease in MC at 30%. An increase in thickness, TS and EAB at 10% with the following reduction. Excellent UV barrier properties and reduced light transmission.	Antioxidant activity: DPPH and FRAP increase, prevention of packaged walnuts oxidation.	[190]
Purple onion peel extracts	10, 20 or 30% (*v*/*v*)	sodium alginate/glycerol (2 g:40% (*w*/*w* of alginate))	An increase in thickness and WVP. A decrease in WS, TS, EAB and transparency. Changes in color.	Antioxidant activity: ABTS and FRAP.	[191]
Corn husk fiber	1.0, 3.0, 5.0 or 8.0 g/100 g of LMP	low methyl pectin (LMP)/potassium sorbate/glycerol (5.25 g LMP in 8 g commercial pectin in 250 mL of deionized water:0.09 g:2.4 g) +5.5 mL CaCl_2_ solution (0.5 g)	Homogeneous with furrows. Changes in color. A decrease in density and WVP. WCA demonstrated the hydrophilicity. An increase in thickness. TS decreased and only at 5 g/100 g of the LMP was elevated.	Antioxidant activity: DPPH and FRAP increase.	[192]
Hecan nutshell extract, hazelnut skin extract	0.025, 0.050, 0.075, 0.1% *w*/*v* of 10 mL ethanol	octenyl succinate starch/glycerol (4 g:2 g)	No change in MC, EAB and WVP, and a slight increase in thickness. A decrease in WS, puncture resistance, TS and YM. An increase in WCA corresponded to hydrophobicity. Changes in color. Less transparent, barrier properties against visible light and UV spectra, good biodegradation.	-	[193]
Pomegranate peel extract	1% *w*/*v*	chitosan/glycerol (1 g in 100 mL acetic acid 1% (*w*/*w*):0.75%% (*w*/*w*)) sodium alginate/glycerol (2 g:10% (*w*/*w* of alginate)) + CaCl_2_ solution (2% *w*/*v*)	-	Antioxidant activity: DPPH and FRAP increase, slow down the oxidation of guava and save more ascorbic acid.	[194]
Melanin from watermelon seeds	0.1% and 0.5% (*w*/*w*)	WPI or concentrate/glycerol (10 g:5% (*w*/*w*))	A decrease in WS, EAB, WCA, WVTR, transparency and light transmission. An increase in MC, SI, TS and barrier properties against UV spectra.	Antioxidant activity: ABTS and DPPH, radical scavenging activity elevation.	[195]
Licorice residue extract	10, 30, 50 and 70 g/kg of SPI in 10 mL etanol	SPI/glycerol (6 g:3 g)	Rough and barrier properties against UV spectra. Changes in color. A decrease in WVP and EAB till 50 g/kg of SPI. An increase in TS and ΔE.	Antioxidant activity: ABTS and DPPH increase.	[196]
Tomato pomace oil extract	0.5 and 1% (*w*/*w*)	gelatin/polyethylene glycol (2, 4, 6 g:1% (*w*/*w*))	No changes in EAB. An increase in thickness, WVP and orangeness. A decrease in WS, MC and TS.	Antioxidant activity: DPPH increases.	[197]

Notes: MC—moisture content, WS—water solubility, TS—tensile strength, EAB—elongation at break, EM—elastic modulus, WVP—water vapor permeability, WVTR—water vapor transmission rate, WCA—water contact angle, SD—swelling degree, SI—swelling index, Tg—glass transition temperature, YM—Young’s modulus, ΔE—total color difference, UV—ultraviolet, WPI—whey protein isolate, SPI—soy protein isolate, HPMC—hydroxypropylmethylcellulose, FFS—film-forming solution, TBARS—thiobarbituric acid reactive substances, TBA—thiobarbituric acid, TAC—total antioxidant capacity, PV—peroxide value and TP—total phenolic content.

**Table 2:** Application of bacteriocins in food biodegradable packaging.

Active Agent	Amount of Addition	Biopolymer Matrix	Changes in Film Properties	Changes in Activity	Refs.
Nisin	2000, 3000, 5000 IU/mL	tapioca starch/glycerin/water (5.0:2.5:92.5 in weight); tapioca starch 5% *w*/*w* and 2% *w*/*w*	Decreased stress at break, EM and strain at break. Elevation of WVP, solubility in water and yellow index (YI).	Antimicrobial activity (reduction in CFU) increased in a dose-dependent manner. Bacteriostatic effect against *L. innocua*, reduction in *Zygosaccharomyces bailii* count.	[250,258]
	10,000 IU/mL	6 g of HPMC, 35 mL of ethanol and 65 mL of distilled water	A decrease in TS, YM, transparency and light transmission. An increase in ultimate elongation and WVP.	Antimicrobial activity against *L. ivanoii* (the highest), *L. innocua* (the lowest), *Enterococcus* spp., *S. aureus* and *B. cereus*.	[260]
	100 mg/mL	Na-alginate 2% (*w*/*w*) and plasticizer 0.75% (50% PEG + 50% glycerol)	A decrease in TS and EAB	Antimicrobial activity against *M. luteus* No inhibition of Gram-negative bacteria.	[256]
	51, 102, 153 or 204 (103 IU/g chitosan)	chitosan (1 g in 100 mL of 1% acetic acid solution)	A decrease in TS. An increase in EAB with minimum nisin addition. An increase in WVP and ΔE and a decrease in transparency in a dose-dependent manner.	Antimicrobial activity against *S. aureus*, *L. monocytogenes* (the highest) and *B. cereus* without a dose-dependent manner. No inhibition of *E. Coli* and *S. typhimurium*.	[254]
	2.5, 5.0, 7.5 or 10.0 mg/g chitosan	chitosan (2 g in 100 g of 1% acetic acid solution)	Reduction in TS in dose-dependent manner. An increase in EAB.	-	[261]
	10,000 IU/g	chitosan 1.3% (*w*/*v*), water chestnut starch 1.5% (*w*/*v*), glycerol (1.5 g in 400 mL starch–chitosan solution)	Improved mechanical properties, increase in TS, EAB and WVP. Slight changes in optical properties.	Antimicrobial activity against *S. aureus* and *L. monocytogenes* (the highest). Low inhibition of *E. Coli*.	[259]
	4000, 8000, 12,000 IU/mL	corn zein/glycerol/ethanol (10%:3%:100 mL) gelatin/glycerol/water (10%:3%:100 mL)	An increase in EAB (more remarkable for corn zein film). A decrease in WVP in a dose-dependent manner.	Antimicrobial activity (reduction in CFU) increased in a dose-dependent manner.	[251]
	28, 56, 84 and 112 mg/g gelatin	gelatin 2% (*w*/*w*), glycerol (30 g/100 g)	Changes in mechanical properties depended on nisin content. Reduction in TS, EM at high nisin content, increase in EAB. Slight increase in WVP. No influence on color parameters and opacity.	Antimicrobial activity against *S. aureus* and *L. monocytogenes* without a dose-dependent manner.	[255]
	0.12 g/100 g of FFS	gelatin (3 g/100 g of FFS), glycerol (25 g/100 g of protein)	Reduction in EAB. A slight increase in TS. Insignificant change in color parameters, light transmission and transparency of gelatin films. A slight decrease in WVP.	No inhibition of *P. Aeruginosa*. Antimicrobial activity in descending order against *S. aureus*, *B. cereus* and *E. coli*.	[257]
Nisin Natamycin	0.0068 g nisin/100 g FFS; 0.027 g natamycin/100 g FFS	for films with nisin/tapioca starch/glycerol/water (3:1:56, in weight); for films with natamycin/tapioca starch/glycerol/water (1.8:1:32.5, in weight)	Nisin addition led to a decrease in stress at break, firmness at break and an increase in the strain at break. No influence on WVP. Changes in color of films with nisin, while the effect of natamycin on optical parameters was less pronounced.	-	[262]
Pediocin	25% and 50% (*w*/*w*) of cellulose weight	cellulose acetate	Affected both the mechanical properties and microstructure of the films: increased thickness, force in rupture and surface roughness.	Antimicrobial activity against *L. innocua*, slight effect against *Salmonella* sp.	[252]
	30, 40 and 50% (*w*/*w*)	cellulose acetate/acetone (1:10 *w*/*v*)	Increased and then decreased the load at break. An increase in thickness in a dose-dependent manner. An increase in WVP at 50% pediocin.	Antimicrobial activity against *L. innocua* and *L. monocytogenes*.	[253]
Enterocin	5 mL (3200 AU mL^−1^)	agar (5%, 2.5%, 1.25% and 0.8%, *w*/*v*), glycerol (20% (*w*/*w*) of agar)	-	Antimicrobial activity against *L. monocytogenes*.	[249]
	100 mL enterocin whey solution	gelatin (6 g), glycerol (2 g)	Barrier properties (WVTR, WVP, LTR and LP) and mechanical properties (TS and EAB) were not significantly affected.	Antimicrobial activity against *L. monocytogenes*.	[247]

Notes: EM—elastic modulus, TS—tensile strength, YM—Young’s modulus, WVP—water vapor permeability, WVTR—water vapor transmission rate, EAB—elongation at break, YI—yellow index, ΔE—total color difference, LTR—limonene transmission rate, LP—limonene permeability, CFU—colony-forming units, HPMC—hydroxypropyl methylcellulose, FFS—film-forming solution and PEG—polyethylene glycol.

**Table 3:** Animal biologically active agents used in food biodegradable packaging.

Active Agent	Amount of Addition	Biopolymer Matrix	Changes in Properties	Changes in Activity	Refs.
Lysozyme	0.143 g/g of low methoxyl (LM) pectin	LM pectin (aqueous solution, 30 g/L) and glycerol (0.2 g/g of LM pectin)	Increased the YM, a slight decrease in EAB.	-	[279]
	2, 4, 6, 8 and 10% *w*/*w* of starch	starch (4% *w*/*v*) and glycerol (30% based on the weight of starch)	Increased TS and YM at 8%, while at 2% and 4%, TS was decreased. The WVP and thickness were increased in a dose-dependent manner.	Antimicrobial activity against *M. lysodeikticus* increased in a dose-dependent manner.	[280]
LPS	5% (*v*/*v*)	chitosan (1.5% *w*/*v*), acetic acid (1% *v*/*v*) and glycerol (0.75 mL/g of chitosan)	-	Antimicrobial activity against *S. putrefaciens*, *P. fluorescens*, psychrotrophic and mesophilic bacteria.	[295]
	1, 2, 3, 4, 5 and 10% (*w*/*w*)	defatted soybean meal (DSM, water suspension, 10% *w*/*v*), glycerol at 20%, 30% or 50% (*w*/*w* DSM) and polysorbate-20 (1% *w*/*w* of DSM)	Decreased TS and YM. No effect on EAB and WVP.	Antimicrobial activity against *S. typhimurium*.	[296]
	0, 1.25, 2.5, 5 and 7.5% (*v*/*v*)	whey protein (aqueous solution, 10% *w*/*v*)/glycerol (1:1)	-	Antimicrobial activity against *S. putrefaciens* and *P. fluorescens*.	[297]
	5% (*v*/*v*)	chitosan (0.5, 1 and 1.5% *w*/*v*), lactic acid (0.875%, *v*/*v*), glycerol (25% p/p of chitosan)	Barrier (WVP) and mechanical properties (TS and EAB) were not significantly affected.	Antimicrobial activity against *X. campestris* and antifungal activity against *C. gloeosporioides* and *L. theobromae*.	[298]
LF	0.5, 1 or 2 mg per disc	chitosan (1.5%, *w*/*v*), acetic acid (1% *v*/*v*) and glycerol (2.5% *w*/*v*)	At 2 mg LF per disc, WVP was increased.	Did not exhibit significant inhibitory effects against *E. coli* and *L. monocytogenes*.	[312]
	1 mL of LF (in PBS)/10 mg bacterial cellulose (BC)	BC nanofibers	The WVP was not altered. A decrease in YM and TS, a slight decrease in EAB.	Antimicrobial activity against *E. coli* and *S. aureus*.	[313]
OTF	25 mg OTF per g of κ-carrageenan	κ-carrageenan (aqueous solution, 2% *w*/*w*), 1.5% plasticizer (50% polyethylene glycol + 50% glycerol)	-	Slight antimicrobial activity against aerobic bacteria and *E. coli* was increased synergistically in the presence of 5 mM EDTA.	[314]
	0, 5, 10, 15% (*w*/*w*) per biopolymer	fish gelatin (2 g) was dissolved in 10 mL of food-grade acetic acid	Reduction in WCA and TS.	Antimicrobial activity against *E. coli* (mostly), *F. psychrophilum*, *S. putrefaciens* and *P. florescens* at 15% OTF.	[315]
LF + Lysozyme	5% (*w*/*w*) of LF and 5% (*w*/*w*) of lysozyme	carboxymethyl cellulose (CMC)	-	Reduction in total aerobic count (TAC).	[316]
Gelatin hydrolysates (GHs)	1 mg/mL of GH	4% (*w*/*v*) fish gelatin (FG) solution	Lowered the viscosity of solution. A decrease in gelling and melting points.	The shelf life of coated shrimp samples was extended by three days when stored at 4 °C due to a reduction in free fatty acid content, total volatile base nitrogen, lipid oxidation and carbonyl content.	[317]
Casein hydrolysates (CHs)	0.05 to 0.3%, *w*/*v*	whey protein concentrate (WPC) powder (5% *w*/*v*), sorbitol (2.5% *w*/*v*), glucomannan (0.25% *w*/*v*), calcium chloride (0.125% *w*/*v*), carboxymethyl cellulose (0.25% *w*/*v*)	-	A decrease in coliform counts with increasing CH concentration up to 0.2%. The antioxidative persistence was enhanced.	[318]

Notes: YM—Young’s modulus, EAB—elongation at break, TS—tensile strength, WVP—water vapor permeability, LPS—lactoperoxidase system, LF—lactoferrin, OTF—ovotransferrin, GH—gelatin hydrolysates, CH—casein hydrolysates, LM pectin—low methoxyl pectin, DSM—defatted soybean meal, CMC—carboxymethyl cellulose, FG—fish gelatin, WPC—whey protein concentrate, BC—bacterial cellulose, TAC—total aerobic count, EDTA—ethylenediamine tetraacetic acid and PBS—phosphate-buffered saline.

**Table 4:** Organic nanoparticles used in food packaging.

Nanomaterial	Amount of Addition	Biopolymer Matrix	Changes in Film Properties	Changes in Activity	Refs.
CNFs	10 wt.%	0.2 wt.% solids consistency PLA	An increase in the YM and TS, and the WVP was reduced.	-	[379]
	1–10 wt.%	xylan/alginate/glycerol (3.6 g:3.6 g:0.8 g)	An increase in the TS, YM and WVP.	-	[380]
	20–100%, *w*/*w*	starch (4%, *w*/*v*)/chitosan (1%, *w*/*v*) (1:1) + 20% (*w*/*w*) of glycerol based on the dry weight of starch and chitosan	The light, oxygen and water vapor barrier capacities were reinforced. With a high concentration of CNFs (≥60%), the rigidity of the films was enhanced.	Stronger antimicrobial properties in a dose-dependent manner.	[381]
	0.032–0.026 g	starch/glycerol/citric acid/sodium hypophosphite (3.0 g:0.9 g:0.15 g:0.075 g)	At 5% *w*/*w,* showed better mechanical properties and an increase in the TS.	-	[382]
	2.5%, 5% and 7.5% *w*/*w* of gelatin	gelatin powder (3.5% *w*/*w*) and 40% glycerol (based on gelatin)	At 5%, boosted YM and TS, but decreased EAB, WVP and moisture absorption (MA).	-	[383]
	2.5, 5, 7.5 and 10%	WPI (10% *w*/*v*) and glycerol (6% *w*/*w*)	At 7.5%, improved water resistance (WVP, WS, MA and MC), increased TS and YM, while EAB decreased. The addition of CNFs at high concentrations reduced TS and YM, while EAB increased.	-	[384]
	5% *w*/*w*, based on the dry weight of SPI	SPI (6% *w*/*v*) and glycerol (60% *w*/*w*, based on the dry weight of SPI)	Enhanced the mechanical properties and TS.	-	[385]
CNCs	5 wt.% on a chitosan basis	2% wt./v chitosan	Significantly improved TS, YM and EAB, but decreased transparency		[386]
	3 wt.% with respect to chitosan 5 wt.% based on the mass of alginate	chitosan (1.5 wt.%) dissolved in 1 *v*/*v*% lactic acid together with 30% glycerol (based on biopolymer mass) alginate (1.5 wt.%) and glycerol (30 wt.% with respect to biomass)	The modification of CNCs prior to their integration positively influenced WCA, WVTR and mechanical properties, which was especially noticeable in environments with high humidity (RH 75%). The best performance was observed in films with incorporated pristine CNCs.	-	[387]
	1, 3 or 5 wt.%	2 g of kappa-carrageenan in 100 mL of distilled water with addition of glycerol (25 wt.% on biopolymer solid base)	The TS and YM increased in a dose-dependent manner. The oxygen barrier property was best enhanced at 3.0 wt.%.	-	[388]
	1, 3, 5 and 10 wt.%	sodium alginate (1 *w*/*v*) and glycerol (0.25 g/g of alginate)	WS and WVP decreased in a dose-dependent manner. TS increased with increasing CNCs content from 0 to 5%. A decrease in film transparency.	-	[389]
	suspension (0.1% *w*/*w*) in 1–10% *w*/*w* in the dry chitosan-based nanocomposite film	chitosan (1%, *w*/*v*) in 2% aqueous acetic acid solution	An increase in TS and a reduction in WVP at 5%.	-	[390]
	0.5% (*w*/*v*)	SPI/glycerol (6 g:2.5 g)	Elevated the TS, barrier ability and thermal stability.	-	[391]
	CNCs (2.0% *w*/*v*) at different mass ratios: 1, 2, 3, 4, 5, 6 and 7 wt.%	2.0% (*w*/*v*) collagen solution	Improved the light transmittance, barrier property, TS, YM and thermostability; at 4 wt.%, the WVP reached the minimum.	-	[392]
	0, 2, 5 and 8 wt.%	WPI (5% by weight) and glycerol (50% solid)	An increase in strength and barrier properties.	-	[393]
	0, 2.5, 5.0, 7.5 and 10.0 wt.% (based on gluten weight)	surfactant (0.2 wt.%), glycerol (1.3 wt.%) and wheat gluten (5 wt.%)	Mechanical properties improved significantly with low loadings of CNCs. Elevated WVP and melting point (at 10 wt.%).	-	[394]
	0.44–3%	3% corn starch, 20% glycerol and gelatin (6–14%)	Higher concentrations of gelatin and CNCs resulted in improved mechanical properties.	-	[395]
BNC	in situ	addition of 2% (*w*/*v*) alginate during the static culture of *Gluconacetobacter* *sucrofermentans* B-11267 for 5 days at 28 °C and crosslinked by an aqueous solution of 5% CaCl_2_ for 3 h	Wet nanocomposite demonstrated higher TS, while dried one demonstrated elongation.	-	[396]
		chitin nanofiber was added in the culture medium for the growth of *G. xylinus* at a concentration of 1 mg/mL, 14 days of incubation	Improved the mechanical strength and barrier property.	-	[397]
		starch (4%) as the matrix of the nanocomposite, glycerol (2%) as the plasticizer, citric acid (0.24%) as the crosslinking agent and NaH_2_PO_4_ (0.12%) as the catalyst of the chemical crosslinking inoculated with a recently isolated strain of *Glucanacetobacter medellinensis* (15%)	Improvement of the water and thermal stability, an increase in TS and YM.	-	[398]
	ex situ	The BNC sheets were immersed in the 1% acetic acid solution, in which 1% chitosan was dissolved	Improved mechanical properties, water holding capacity (WHC) and water release rate (WRR).	-	[399]
	0%, 5% and 10% (based on CH dry weight)	2% chitosan (CH) solution and 30% glycerol (based on the CH dry weight)	Lowered the high WS, improved the mechanical properties.	-	[400]
	0%, 1%, 2%, 3% and 4% wt., based on the weight of KGM	1% (*w*/*v*) konjac glucomannan (KGM) and 30% glycerol (*w*/*w*, based on the KGM)	Improved physical and barrier properties. An increase in TS. EAB was increased and then decreased.	-	[401]
	1, 5 and 10% *w*/*w*, on starch dry basis	Potato starch powder (2% *w*/*v*) and glycerol (20% *w*/*w*, on starch dry basis)	An increase in strength (YM and TS), resistance to both MA and WS. Reduced elasticity.	-	[402]
	1.1, 1.6, 1.8 and 2.0% (based on protein weight)	Ten milliliters of the DDGS (buckwheat distiller’s dried grains) protein solution (5%) was mixed with sorbitol (0.25 g, half of the weight of the protein)	1.8% and 2.0% improved the properties of the biocomposite films, promoted the mechanical properties and WVP.	-	[403]
ChNPs	1.0, 3.0 and 5.0 wt.%	PLA	Slightly decreased the thermal stability, improved the elongation and the impact strength, but decreased the TS.	-	[404]
	0.2, 0.5 and 0.8 in wt.%	2.0 wt.% CMC	Improvement of thermal and mechanical properties.	-	[405]
	0, 1, 2, 4, 6 and 8 wt.% based on the amount of potato starch	5 g of potato starch and glycerol (1.5 g)	Improved the effect on the TS, storage modulus, glass transition temperature, water vapor barrier and thermal stability, higher ChNP loads (8 wt.%) resulted in the aggregation in the composites.	-	[406]
	Formation of particles in starch hydrogels was observed by changes in turbidity	Two portions of 5% (*w*/*w*) starch hydrogel, one of which contains dissolved chitosan, and the second contains salt magnesium sulfate (crosslinker) mixed in a volume ratio of 1:1 and added 1% (*w*/*w*) glycerol (20% by weight of starch)	Increased the elongation and flexibility, lower vapor permeability.	-	[407]
	0, 5, 10, 15, 20% *w*/*w*	3% *w*/*w* tapioca starch and 25% *w*/*w* glycerol of the dry starch solid weight	-	Antimicrobial activity against *B. cereus*, *S. aureus*, *E. coli* and *S. typhimurium*. More efficient to inhibit the microbial growth in cherry tomatoes (at 15% *w*/*w*).	[408]
	Combined with chitosan at 3:7, 1:1 and 7:3 ratios	3% (*w*/*w*) colloidal film chitosan blends	Positively affects film mechanical strength and stiffness.	Antimicrobial activity against *E. coli* and *S. aureus.*	[409]
	5, 10, 15, 20, 25, 30, 35% *w*/*w* of solid starch	3% *w*/*w* solution of tapioca starch and glycerol (25% *w*/*w* of the dry starch solid weight)	5% *w*/*w* enhanced the TS and EAB, reduced thermal stability, WVP and oxygen permeability, increased opacity.	-	[410]
	50 mL of ChNP solution	47 g of cupuassu puree and 3 g of pectin	The WVP decreased, elevated elongation, TS only in pectin films.		[411]
	0, 2, 4, 6 and 8%, *w*/*w*	4 g fish gelatin and glycerol (0.3 g/g gelatin)	Improved the mechanical properties and decreased the WVP.		[412]
	12.5%	Gelatin/glycerol/tapioca starch (10:3:1)	An increase in WVP and EAB, a decrease in TS.	Antimicrobial activity against *S. aureus* and *E. coli.*	[413]
StNPs	0, 1, 3, 6 and 9 wt.%	2% of CMC, 2% of starch mixed in a 2:1 (*w*/*w*) ratio	Enhanced the physico-mechanical properties. Reduction in WPR and oxygen permeability, an increase in the TS.	Antioxidant properties, significantly extended shelf life of chicken to 56 h.	[414]
	0%, 3%, 6%, 9% and 12% on the dry basis of pea starch	pea starch (7.5 g) and glycerol (3.0 g)	The highest TS value at 6%. Elevation of melting temperature and water barrier properties.	-	[415]
	0.0, 0.5, 1.0, 2.0 and 5.0 wt.% (based on maize starch)	7.0 g of maize starch and 3.0 g of glycerol with final solid concentration at 10.0 wt.% (*w*/*v*)	An increase in TS and breaking elongation (at 1%). A decrease in WVP and opacity.	-	[416]
	0, 0.05, 0.15, 0.25, 0.35 and 0.45 g	5.0 g of pea starch and 1.5 g of glycerol	An increase in TS, water vapor barrier and thermostability.	-	[417]
	0.5–10 wt.%, relative to the dry total mass	cassava starch (4 wt.%) and glycerol as plasticizer (2.0 wt.%)	WVTR, TS and EM were influenced by the linear effect of StNP concentration. A decrease in WVP (at 10%) and an increase in traction resistance and EM.	-	[418]
	0.5, 1, 2, 5 and 10%	starch (5%) (*w*/*w*, dry basis), glycerol (2.5%) (*w*/*w*) and vegetable oil (2 g/L)	An increase in thickness, solubility, WVTR and burst strength.	-	[419]
	0, 2, 4, 6 and 8 wt.% on the dry basis of starch	Starch (2 g) added to 30 mL of deionized water to obtain composite solutions	Improved WPR and WCA.	-	[420]
	Peanut protein NPs 0, 0.025, 0.05, 0.1 and 0.2 g	SPI or cornstarch (5 g) and glycerol (1.25 g)	An increase in EAB, improved water vapor barrier and thermostability.	-	[421]
	0, 1, 2, 3, 4 or 5 wt.% was based on the amount of SPI	SPI of desired weight and 33 wt.% glycerol	Enhanced the storage modulus, Tg, TS and YM. A decrease in EAB and water uptake.	-	[422]
	Content in original freeze-dried powders was 1, 2, 3, 4, 8, 12 and 16%	weight ratio of every solid powder (StNPs incorporated into SPI) and glycerol was maintained at 70:30	2 wt.% showed a predominant reinforcing function. Elevated TS and YM.	-	[423]
Protein-based NPs	Zein NPs 5, 10 and 15% *w*/*w* based on starch weight	Potato starch (5 g) and glycerol (35% *w*/*w* based on starch weight)	Improved the mechanical properties of films.	-	[424]
	Zein NPs 0.05, 0.1, 0.5 or 1.0% (*w*/*v*)	3% (*w*/*w*) Methocel A15 Food-Grade-Modified Cellulose	An increase in WCA and TS. A decrease in WPR, capacity to elongate, an initial increase followed by a gradual decrease in YM.	-	[425]
	Zein NPs 0.2, 0.4, 0.8 and 1.2 (*w*/*w* of WPI)	7% (*w*/*v*) solution of whey protein isolate (WPI) and glycerol (60%, *w*/*w* of WPI)	Decreased hydrophilicity, improved moisture barrier and mechanical properties.	-	[426]
	Casein-based NPs 0.02 g/10 mL	5% casein and 1.5% (*v*/*v* basis) of glycerol 2.5% pectin, 1.25% sodium alginate, 0.01% (of whole solution) of CaCl_2_ solution and glycerol (50% *w*/*w* of total polysaccharide)	Improved the water and light barrier properties, TS and thermal properties.	Antimicrobial activity against *E. coli.* due to silver.	[427]

Notes: TS—tensile strength, EAB—elongation at break, YM—Young’s modulus, MC—moisture content, WS—water solubility, WVP—water vapor permeability, MA—moisture absorption, WVTR—water vapor transmission rate, WCA—water contact angle, WHC—water holding capacity, WRR—water release rate, EM—elastic modulus, Tg—glass transition temperature, CNFs—cellulose nanofibrils, CNCs—cellulose nanocrystals, BNC—bacterial nanocellulose, ChNPs—chitosan nanoparticles, StNPs—starch nanoparticles, NPs—nanoparticles, PLA—polylactic acid, SPI—soy protein isolate, WPI—whey protein isolate, KGM—konjac glucomannan, DDGS—buckwheat distiller’s dried grains and CMC—carboxymethyl cellulose.

## Data Availability

Data sharing not applicable.
